# Cohesin-mediated NF-κB signaling limits hematopoietic stem cell self-renewal in aging and inflammation

**DOI:** 10.1084/jem.20181505

**Published:** 2019-01-07

**Authors:** Zhiyang Chen, Elias Moris Amro, Friedrich Becker, Martin Hölzer, Seyed Mohammad Mahdi Rasa, Sospeter Ngoci Njeru, Bing Han, Simone Di Sanzo, Yulin Chen, Duozhuang Tang, Si Tao, Ronny Haenold, Marco Groth, Vasily S. Romanov, Joanna M. Kirkpatrick, Johann M. Kraus, Hans A. Kestler, Manja Marz, Alessandro Ori, Francesco Neri, Yohei Morita, K. Lenhard Rudolph

**Affiliations:** 1 Leibniz Institute on Aging, Fritz Lipmann Institute (FLI), Jena, Germany; 2 RNA Bioinformatics and High-Throughput Analysis, Friedrich Schiller University Jena, Jena, Germany; 3 European Virus Bioinformatics Center (EVBC), Jena, Germany; 4 Matthias Schleiden Institute for Genetics, Bioinformatics and Molecular Botany, Faculty of Biological Sciences, Friedrich Schiller University Jena, Jena, Germany; 5 Institute of Medical Systems Biology, Ulm University, Ulm, Germany; 6 Faculty of Medicine, Friedrich-Schiller-University, Jena, Germany

## Abstract

Organism aging is characterized by increased inflammation and decreased stem cell function, yet the relationship between these factors remains incompletely understood. This study shows that aged hematopoietic stem and progenitor cells (HSPCs) exhibit increased ground-stage NF-κB activity, which enhances their responsiveness to undergo differentiation and loss of self-renewal in response to inflammation. The study identifies *Rad21*/cohesin as a critical mediator of NF-κB signaling, which increases chromatin accessibility in the vicinity of NF-κB target genes in response to inflammation. *Rad21* is required for normal differentiation, but limits self-renewal of hematopoietic stem cells (HSCs) during aging and inflammation in an NF-κB–dependent manner. HSCs from aged mice fail to down-regulate *Rad21*/cohesin and inflammation/differentiation signals in the resolution phase of inflammation. Inhibition of cohesin/NF-κB reverts hypersensitivity of aged HSPCs to inflammation-induced differentiation and myeloid-biased HSCs with disrupted/reduced expression of *Rad21*/cohesin are increasingly selected during aging. Together, *Rad21*/cohesin-mediated NF-κB signaling limits HSPC function during aging and selects for cohesin-deficient HSCs with myeloid-skewed differentiation.

## Abstract (precis)

Chen et al. identify *Rad21*/cohesin as a critical mediator of inflammation/NF-κB–induced differentiation of hematopoietic stem cells (HSCs). Aging-associated increases in inflammation select for HSCs with disrupted or naturally reduced *Rad21*/cohesin expression exhibiting increased self-renewal and myeloid-biased differentiation: two hallmark features of the aging hematopoietic system.

## Graphical Abstract

**Figure:**
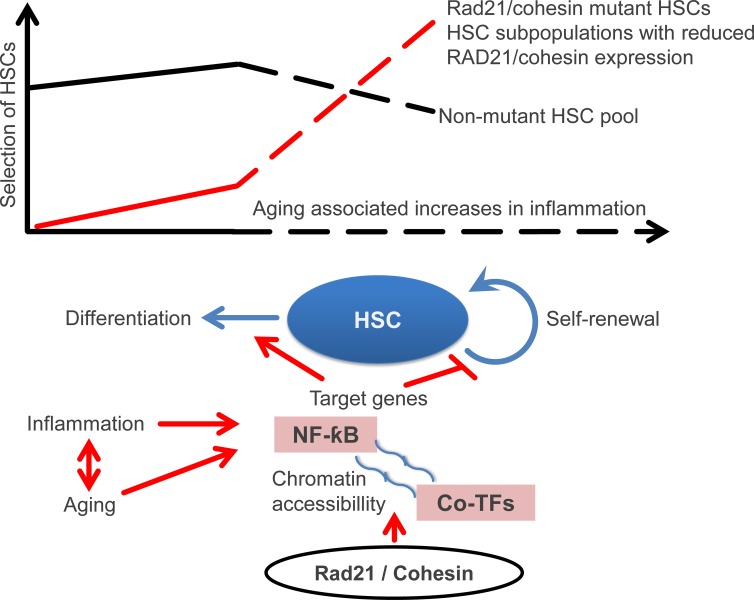

## Introduction

During aging, hematopoietic stem cells (HSCs) exhibit impaired repopulation capacity (Morrison et al., 1996; Sudo et al., 2000), skewed differentiation (Rossi et al., 2005; Cho et al., 2008; Dykstra et al., 2011), and an increase in the selection of clones that carry recurrent mutations (predominantly in genes that regulate epigenetic modification and gene expression; Busque et al., 2012; Genovese et al., 2014; Jaiswal et al., 2014). Mechanistically, the causes of HSC aging remain incompletely understood, but involve a variety of molecular defects, such as alterations in autophagy (Ho et al., 2017), DNA replication (Flach et al., 2014), cell polarity (Florian et al., 2012), and DNA integrity control (Beerman et al., 2014). Causal factors that contribute to the evolution of these molecular defects and to HSC dysfunction during aging involve cell-intrinsic processes, as well as aging-associated alterations in the HSC environment, including the blood circulation (Ju et al., 2007; Song et al., 2010; Ergen et al., 2012; Guidi et al., 2017).

Chronic increases in inflammatory signals have been identified as a hallmark feature of aging and involve a prominent activation of NF-κB target genes, such as IL-6 and TNFα, which are associated with accelerated disease development and mortality during aging (Fagiolo et al., 1993; Brüünsgaard and Pedersen, 2003; Starr et al., 2015). While ground-stage activity of NF-κB signaling is required for the maintenance of HSCs (Fang et al., 2018), studies on genetic mouse models of inflammation and DNA damage showed that the chronic induction of inflammatory signals can contribute to the decline of HSC function (Ju et al., 2007; Song et al., 2010; Franceschi and Campisi, 2014; Walter et al., 2015; Kovtonyuk et al., 2016; Pietras et al., 2016) and may also be amenable to therapeutic intervention to improve tissue maintenance in aging (Yu et al., 2014).

Experimental induction of inflammation/NF-κB signaling leads to an activation and expansion of hematopoietic stem and progenitor cells (HSPCs), enhanced mobilization of HSPCs, and a shift toward myeloid differentiation, required for the mounting of immune responses (Nagai et al., 2006; Jaiswal et al., 2009; Zhao et al., 2014). During aging, an increase in NF-κB activation has been reported in HSCs (Chambers et al., 2007), but mechanisms and consequences of NF-κB activation on the maintenance and function of HSPCs in response to aging and inflammation remain to be delineated. Here, we show that aging HSCs exhibit an increase in ground-stage activity of NF-κB signaling and a failure to down-regulate inflammation/NF-κB and differentiation signals in the resolution phase after inflammatory stimulation. These changes associate with an enhanced sensitivity of HSCs from old versus young mice to undergo differentiation and loss of self-renewal in response to inflammatory stimuli. The study defines a new role of *Rad21*/cohesin as a mediator of NF-κB signaling, in limiting the functional reserve and self-renewal of HSCs during aging and inflammation, which in turn increases selection of cohesin mutant HSCs with impaired and myeloid-skewed differentiation.

## Results

### Aging HSCs exhibit increased ground-stage activity of NF-κB and enhanced sensitivity to inflammation/NF-κB–induced loss of self-renewal

**Figure 1.**
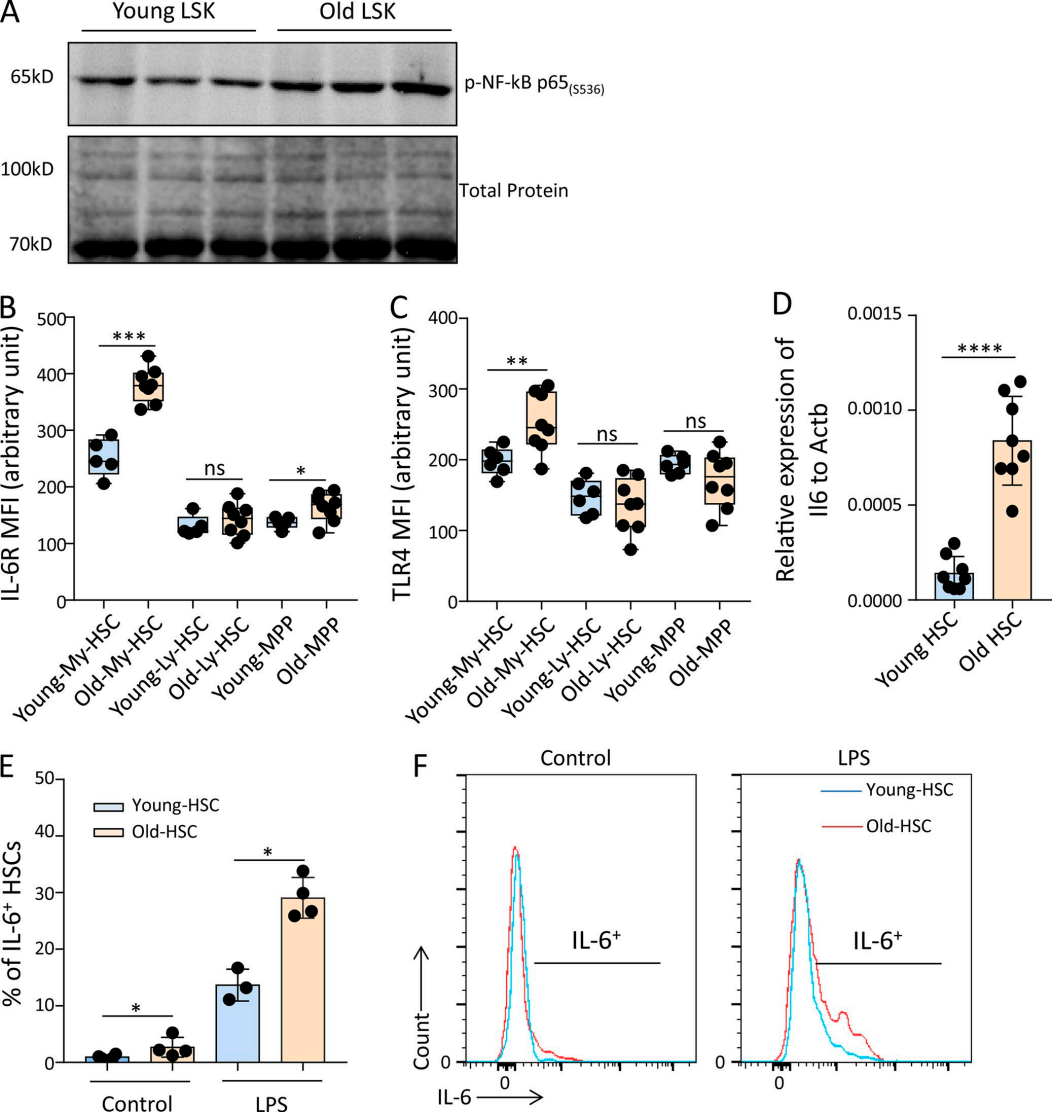
**Aging increases the ground-stage activity of NF-κB signaling in HSPCs. (A)** Representative Western blot showing the level of phospho-NF-κB p65 (Ser536) in LSK cells from young (2–3 mo old) and old (24 mo ­old) mice (*n* = 3 mice per pool per lane for each experiment, *n* = 2 independent experiments, one of the two experiments is shown; the other experiment shows a similar result). **(B and C)** Mean fluorescence intensities (MFI) determined by FACS for IL-6R and TLR4 expression on freshly isolated My-biased HSCs, Ly-biased HSCs, and MPPs from young (2–3 mo old) and old mice (22–24 mo old). The box plots represent the interquartile range (25–75%), with the median; whiskers correspond to min and max values. The dots indicate individual mice (in total, *n* = 5–8 mice per group were analyzed in *n* = 2 independent experiments). My-biased HSC: CD150^hi^CD34^−^LSK; Ly-biased HSC: CD150^lo^CD34^−^LSK; MPP: CD34^+^LSK. **(D)** mRNA expression of *Il6* relative to *Actb* was analyzed in freshly isolated HSCs from young (2 mo old) and old (24 mo old) mice (in total, *n* = 8 mice per group were analyzed in *n* = 2 independent experiments). HSC: CD150^+^CD34^−^LSK. **(E and F)** Young (3 mo old) and old (24 mo old) wild-type mice received an i.p. injection of LPS (1.5 mg/kg) and were sacrificed 3 h later. c-Kit^+^–enriched BM cells were isolated and cultured for 4 h with secretion inhibitor (Brefeldin A). The level of IL-6 in the HSC population was measured by FACS (*n* = 3–4 mice per group were used in total in *n* = 2 independent experiments). **(E)** The histogram depicts the percentages of IL-6–positive HSCs of the indicated age groups. **(F)** Representative FACS profiles showing the level of IL-6 in indicated groups.**(B–E)** Statistical significance was assessed by using the Welch’s *t* test after log transformation (B–D) or with the two-way ANOVA followed by Tukey’s multiple comparison test on logit-transformed data (E). All data represent mean ± SD; *, P < 0.05; **, P < 0.01; ***, P < 0.001; ****, P < 0.0001; ns, not significant.

Increased inflammation has been identified as a hallmark feature of organism aging and as a driving factor for the evolution of multiple aging-associated diseases (Ju et al., 2007; Song et al., 2010; Walter et al., 2015; Kovtonyuk et al., 2016; Pietras et al., 2016). The availability of data describing the increase in inflammation/NF-κB signaling in the aging hematopoietic system is sparse, but an increase in nuclear expression of p65/NF-κB was reported in aging HSCs (Chambers et al., 2007). In line with this observation, we detected an increase in phospho-p65 in Western blot analysis on freshly isolated HSPCs (lineage^−^, Sca-1^+^, c-Kit^+^ cells; LSK cells) from old (24 mo old) compared with young (2–3 mo old) mice (Fig. 1 A). FACS analysis was used to monitor expression levels of inflammatory receptors on freshly isolated, highly purified HSCs from young and old mice. In HSCs of 22–24-mo-old mice compared with 2–3-mo-old mice, a significant increase in the expression of IL-6 receptor (IL-6R) and TLR4, two bona fide NF-κB targets contributing to induction of inflammation/NF-κB signaling (Caiello et al., 2014; Wan et al., 2016; Liu et al., 2017), was observed (Fig. 1, B and C). In contrast, multipotent progenitor cells (MPPs; CD34^+^LSK) showed little or no increase in these inflammatory receptors (Fig. 1, B and C). The increase in inflammatory receptor expression in HSCs from old mice compared with young mice was associated with elevated expression of *Il6* mRNA (a prominent NF-κB target cytokine encoding gene) in freshly isolated HSCs from old compared with young mice (Fig. 1 D). HSCs from old versus young mice also exhibited an increase in IL-6 protein production in response to LPS stimulation (Fig. 1, E and F). Together, these results provided evidence for elevated ground-stage activity of NF-κB signaling in freshly isolated aged HSCs.

To test whether increases in ground-stage NF-κB activity would alter the responsiveness of HSCs to inflammatory signals or the fate of HSCs from old compared with young mice, NF-κB reporter mice were used (Krieger et al., 2018). These mice express EGFP under a promoter containing a repeat element for NF-κB binding, thus facilitating the analysis of the percentage of living cells that exhibit active NF-κB signaling at a given time. This allowed us to study consequences of endogenous activation of NF-κB signaling in steady-state hematopoiesis comparing HSPCs with active NF-κB (GFP^+^) with NF-κB–negative HSPCs (GFP^−^) from young (3 mo old) and old (24 mo old) NF-κB reporter mice.

**Figure 2.**
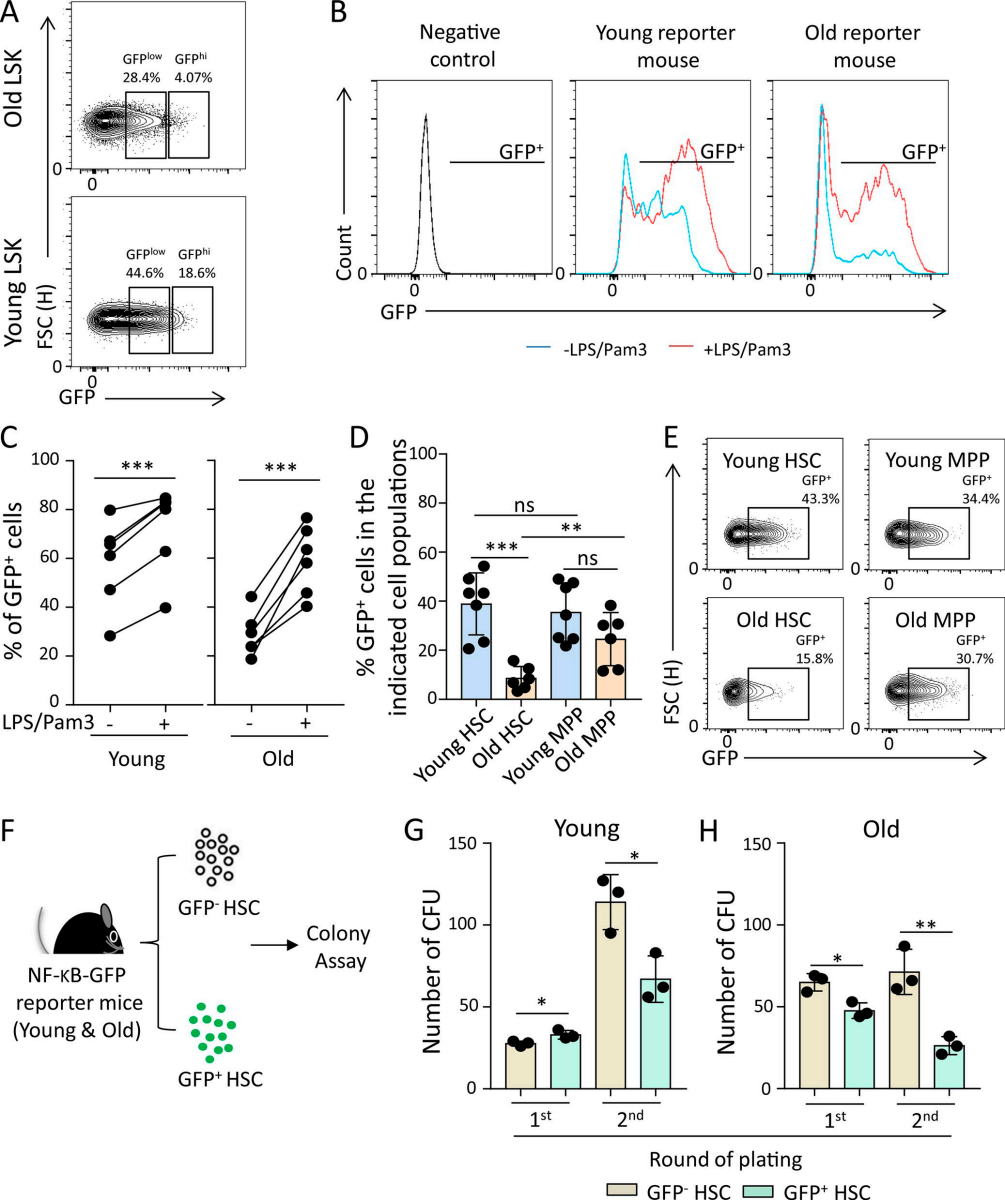
**NF-κB activation in steady-state hematopoiesis phenotypically correlates with enhanced differentiation of HSPCs in aging mice. (A)** Representative FACS plots showing percentages of GFP^low^ and GFP^hi^ cells in LSK cells of the indicated groups. **(B and C)** Freshly isolated LSK cells from young (3 mo old) and old (24 mo old) NF-κB reporter mice were cultured with or without LPS/Pam3 treatment for 8 h. The percentages of GFP^+^ cells (NF-κB–activated cells) were determined by FACS. **(B)** Representative FACS profiles for GFP expression in the indicated groups. **(C)** The chart shows percentages of GFP^+^ cells in stimulated LSK cells compared with nonstimulated LSK cells of the indicated groups (in total, *n* = 6 mice per group were analyzed in *n* = 2 independent experiments). The dots indicate pairs of aliquots of LSK cells from the same individual mice exposed to the indicated treatments. Statistical significance was assessed by paired *t* test after logit transformation of the data. **(D)** The percentage of GFP^+^ cells in the indicated hematopoietic populations from young (3 mo old) and old (24 mo old) NF-κB reporter mice was determined by FACS (in total, *n* = 7 young mice and *n* = 6 old mice were analyzed in *n* = 2 independent experiments). **(E)** Representative FACS plots showing percentages of GFP^+^ cells in HSCs and MPPs of the indicated groups. Statistical significance was assessed by Welch’s *t* test after logit transformation of the data. **(F–H)** CD150^+^CD34^−^LSK cells were sorted from the BM of young (2–3 mo old) and old (24 mo old) NF-κB reporter mice and serially plated in methylcellulose medium at 12-d intervals (first plating: 500 CD150^+^CD34^−^LSK cells/triplicate; second plating: 5,000 CD45^+^DAPI^−^ cells/duplicate). **(F)** Schematic diagram of the experiment. **(G and H)** The histograms show the absolute number of colonies in the indicated groups and round of plating (in total, *n* = 3 mice per group were analyzed in two independent experiments, *n* = 2–3 technical repeats per mouse). Statistical significance was assessed after log transformation by Welch’s *t* test. All data represent mean ± SD; *, P < 0.05; **, P < 0.01; ***, P < 0.001; ns, not significant. FSC, forward scatter.

Unexpectedly, freshly isolated HSPCs from old mice exhibited a lower percentage of reporter activity (Fig. 2 A). When exposed to LPS plus Pam3CysSerLys4 (Pam3), reporter activity was induced in HSPCs from both young and old mice (Fig. 2, B and C), and the absolute level of LPS/Pam3-induced reporter activity was similar in HSPCs from young and old mice (72.28 ± 17.85% in young mice vs. 59.22 ± 14.14% in old mice; P = 0.1501). Together, these data indicated that HSPCs from young and old mice respond similarly in inducing NF-κB reporter activity, but freshly isolated HSPCs from old mice have a lower starting level of reporter activity. A possible explanation for these findings was that HSPCs from old mice are quickly depleted in response to NF-κB activation and the aforementioned increase in ground-stage activity of NF-κB signaling during aging was not depicted in reporter activity, possibly because it was below the detection limit of the reporter. To directly test whether the induction of endogenous NF-κB signaling would affect the self-renewal of HSPCs in an age-dependent manner, we analyzed the percentage of NF-κB–positive cells (GFP^+^) in the fraction of HSCs versus MPPs in freshly isolated bone marrow (BM) cells from 3- and 24-mo-old, untreated NF-κB reporter mice. In old compared with young mice, the percentage of NF-κB–positive HSCs was significantly diminished, whereas the percentage of NF-κB–positive MPPs did not show a significant difference among the age groups (Fig. 2, D and E). Moreover, there was a significant reduction in the percentage of NF-κB–positive HSCs versus MPPs in old mice detectable, but there was no difference in the percentage of NF-κB–positive HSCs versus MPPs in young mice (Fig. 2 D).

To further validate the negative impact of endogenous NF-κB activity in steady-state hematopoiesis on the self-renewal potential of HSCs from aging mice, GFP^+^ and GFP^−^ HSCs (CD150^+^CD34^−^LSK) were purified from young (3 mo old) and old (24 mo old) NF-κB reporter mice and were serially plated in methylcellulose to determine self-renewal/expansion potential of HSC-derived cells harboring CFU potential (Fig. 2, F–H). In freshly isolated HSCs from young mice, NF-κB activity was associated with an increase in the CFU potential in the first round of plating (Fig. 2 G). In the second round of plating, NF-κB–negative HSC–derived cells showed a strong expansion of cells harboring CFU potential. This increase in CFUs in the second round of plating was reduced in NF-κB–positive HSCs from young mice (Fig. 2 G). In HSCs from old mice, NF-κB activity was associated with a reduced CFU potential already in the first round of plating (Fig. 2 H). Moreover, the magnitude of the impairment in CFU potential in the second round of plating of cells derived from NF-κB–positive versus NF-κB–negative HSCs was higher in cells derived from old mice compared with cells derived from young mice (Fig. 2, G and H; 62.4 ± 9.71% reduction in old vs. 41.3 ± 6.85% reduction in young; P = 0.0453). Together, these data indicated that endogenous activation of NF-κB signaling has a stronger effect on impairing the self-renewal capacity of HSCs from aged mice compared with HSCs from young mice.

### *Rad21*/cohesin mediates NF-κB–induced transcriptional responses and differentiation of HSPCs

**Figure 3.**
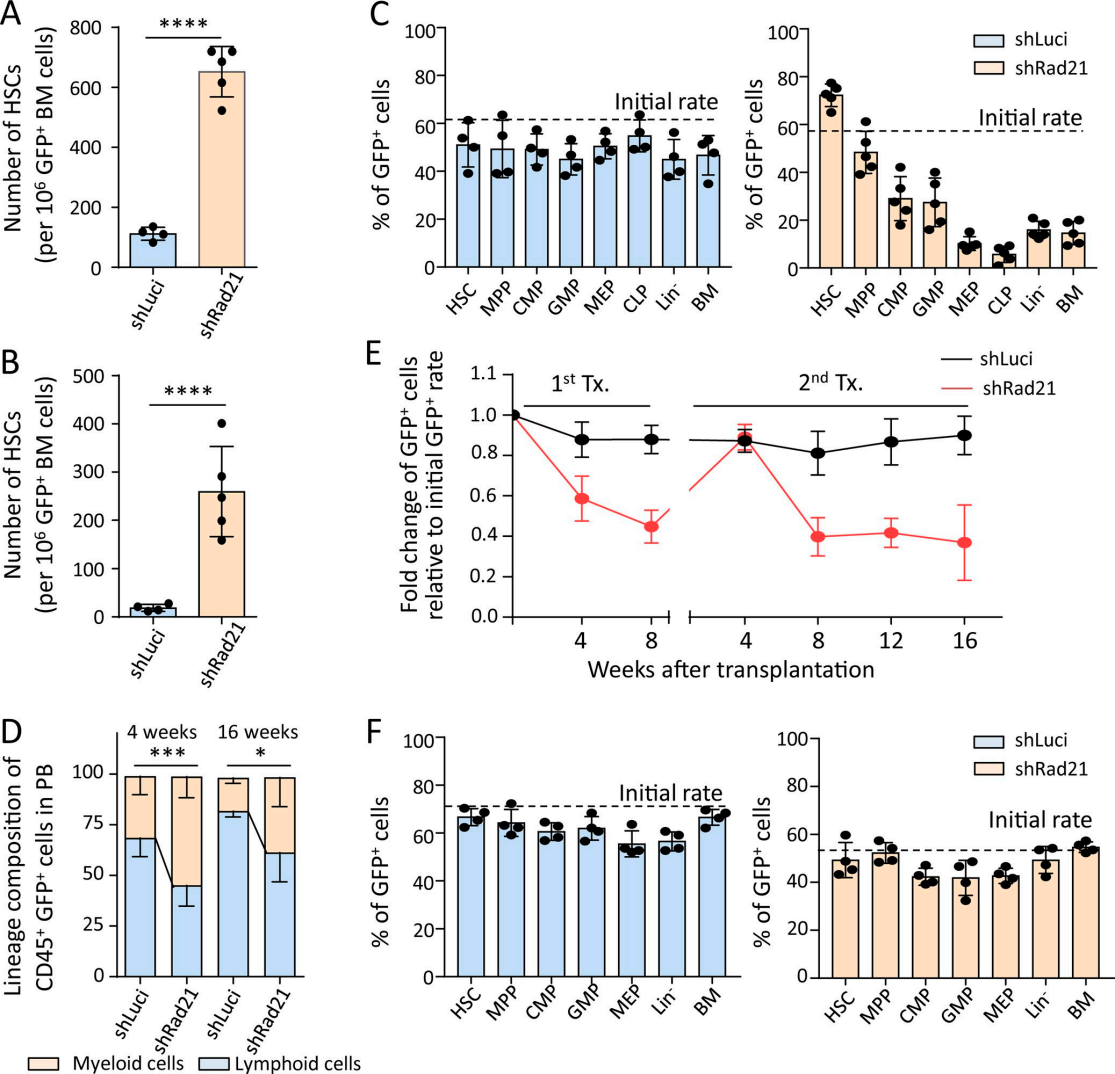
***Rad21* deficiency increases self-renewal and leads to impaired and myeloid-skewed differentiation of HSPCs. (A–D)** Freshly isolated LSK cells were infected with an shRNA against *Rad21* shRad21 or a control virus (shLuci) in culture and transplanted into lethally irradiated mice together with noninfected LSK cells from the same culture. **(A and B)** The number of HSCs (GFP^+^CD150^+^CD34^−^LSK cells) derived from infected LSK cells in the primary recipients (A) and the secondary recipients (B) that were transplanted with total BM cells of the primary recipients at 4 mo after the first transplantation. The dots represent individual recipient mice (*n* = 4–5 mice per group per experiment, *n* = 3 independent experiments, one of the three repeat experiments is shown, the other experiments showed similar results). Statistical values were calculated by Welch’s *t* test after log transformation. **(C)** The chimerism of GFP^+^ cells from the primary recipients was analyzed by FACS at 4 mo after transplantation in the indicated populations. HSC: CD150^+^CD34^−^LSK; MPP: CD34^+^LSK; CMP: common myeloid progenitor (Lin^−^c-Kit^+^Sca-1^−^CD34^+^FcγR^−^); GMP: granulocyte macrophage progenitor (Lin^−^c-Kit^+^Sca-1^−^CD34^+^FcγR^+^); MEP: megakaryocyte erythrocyte progenitor (Lin^−^c-Kit^+^Sca-1^−^CD34^−^FcγR^−^), CLP: common lymphoid progenitor (Lin^−^c-Kit^low^Sca-1^low^IL-7Ra^+^Flt3^+^), Lin^−^: lineage^−^ cells; BM: total BM cells. *n* = 4–5 mice per group per experiment, three independent experiments, one of the three experiments is shown, the other experiments showed similar results. **(D)** PB cells from the primary recipients were analyzed by FACS. The histogram shows the percentage of myeloid and lymphoid cells in GFP^+^CD45^+^ cells in the PB at the indicated time points after transplantation (*n* = 4–5 mice per group for each experiment, *n* = 3 independent experiments, one of the three experiments is shown; the other experiments showed similar results). Statistical significance for myeloid cells was assessed by Welch’s *t* test after logit transformation of the data. **(E and F)** LSK cells from young (2 mo old) wild-type mice were transduced with shLuci or shRad21 virus followed by transplantation into 2–3-mo-old recipients. 8 wk after transplantation, recipient mice were sacrificed and total BM was transplanted into secondary recipients. 4 wk after secondary transplantation, BM cells were analyzed to investigate the chimerism of shLuci- or shRad21-LSK cells in BM. Chimerism of shLuci- or shRad21-infected LSK cells in PB was monitored every 4 wk after each round of transplantation. **(E)** The data show the relative change of GFP^+^ cells in PB from the recipients at indicated time points after transplantation. The initial transduction efficiency was 71% for shLuci-LSK and 54% for shRad21-LSK (*n* = 5 recipient mice per group, per experiment in the primary transplantation, *n* = 10 recipient mice per group, per experiment in the secondary transplantation, *n* = 2 independent experiments, one of the two experiments is shown; the other experiment showed a similar result). **(F)** The histograms depict the chimerism of GFP^+^ cells in the indicated populations of the secondary recipients at 4 wk after transplantation. The dots represent individual mice (*n* = 4 mice per group for each experiment, *n* = 2 independent experiments, one of the two experiments is shown, the other experiment showed a similar result). Data in all panels of this figure depict mean ± SD; *, P < 0.05; ***, P < 0.001; ****, P < 0.0001.

To determine candidate genes that may contribute to age-related changes in NF-κB–mediated gene expression, an shRNA screen was conducted to identify genes that influence differentiation of HSCs in mice that were exposed to ad libitum (AL) diet or dietary restriction (DR), an intervention known to inhibit differentiation of HSPCs by suppressing the expression of cytokines that are known NF-κB targets, such as IL-6 (Tang et al., 2016). This screen revealed that *Rad21* knockdown strongly impairs differentiation of transplanted HSPCs in recipients exposed to DR, but to a lesser extent in AL-fed recipients (Fig. S1, A and B), known to express higher levels of differentiation inducing cytokines in blood serum compared to DR-exposed mice (Tang et al., 2016). Aside the partial rescue of the compromised differentiation capacity of *Rad21* knockdown cells in AL-fed mice compared with DR-fed mice and in line with previous studies on unperturbed hematopoiesis (Mullenders et al., 2015; Viny et al., 2015), knockdown of *Rad21* resulted in a robust enhancement of self-renewal of HSCs in serial transplantation experiments in AL-fed mice (Fig. 3, A and B; and Fig. S1, C and D). While control cells (shLuciferase [shLuci]) did not show changes in the differentiation capacity of HSPCs compared with cotransplanted, noninfected (GFP negative) cells (Fig. 3 C, left; and Fig. S1 E), *Rad21*-depleted hematopoietic cells exhibited a strong impairment in differentiation into both lymphoid and myeloid lineages (Fig. 3 C, right; and Fig. S1, F and G). Within the population of shRNA-targeted hematopoietic cells (GFP positive), *Rad21* knockdown skewed hematopoiesis toward myeloid differentiation compared with the luciferase control (Fig. 3 D). Of note, impairments in differentiation of *Rad21*-deficient HSPCs were completely abolished at an early time point (4 wk) after secondary transplantation (Fig. 3, E and F). Since NF-κB regulated cytokines are known to reach peak plasma levels at early time points (3–7 wk) after transplantation (Sakata et al., 2001), these results suggested that the inhibitory effects of *Rad21* knockdown on differentiation can be overruled by increases in cytokine levels.

**Figure 4.**
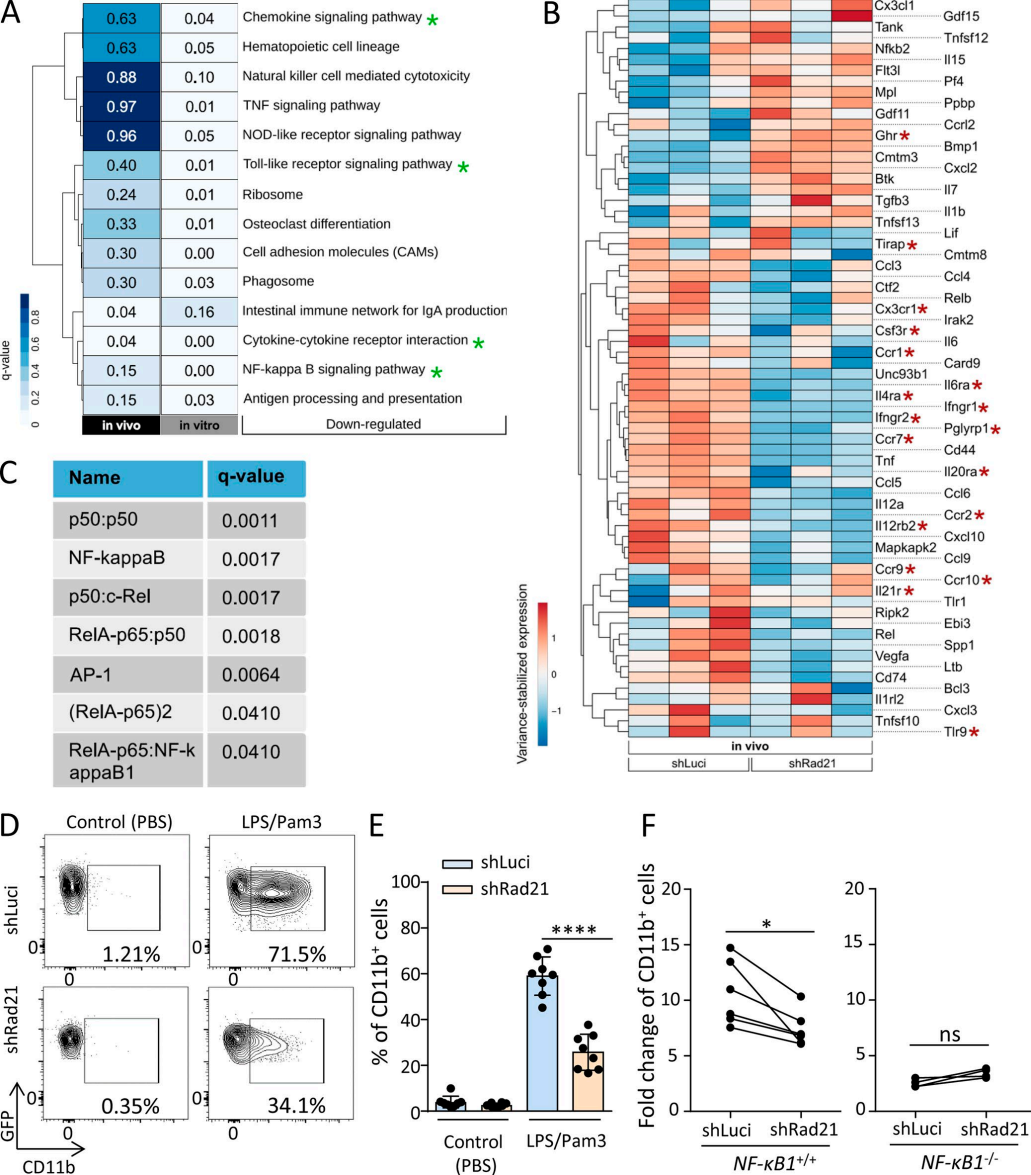
***Rad21* mediates differentiation of HSPCs in response to inflammation through NF-κB–dependent signaling. (A)** Analysis of DEGs in two RNA-seq experiments: (1) on GFP^+^CD48^−^Sca-1^+^ cells purified from cultured LSK cells, 3 d after isolation and lentiviral infection with an shRNA targeting *Rad21* or a control shRNA (luciferase = luci) and (2) on freshly isolated GFP^+^ LSK cells from mice that were transplanted with lentivirally infected LSK cells (shRNA-Rad21 or shRNA-luci), 4 mo after transplantation. KEGG pathway analysis shows the 14 most down-regulated pathways in *Rad21* knockdown cells versus control cells. The analysis did not identify significantly (q < 0.1) up-regulated pathways. Green asterisks highlight NF-κB–related pathways. DEGs in these pathways from the in vivo analysis are depicted in B. **(B)** The heat map shows DEGs based on GO-terms related to the KEGG pathways highlighted in A (in vivo) in freshly isolated *Rad21* knockdown LSK cells (shRad21) versus control (shLuci) LSK cells reisolated 4 mo after LSK cell transplantation. The heat map shows row-scaled and variance-stabilized expression counts for each gene. Red asterisks highlight inflammatory receptor genes. **(C)** The seven top transcription factor complexes, regulating DEGs in the experiment on culture-activated LSK cells (Fig. S2 A), were identified with the help of the GeneTrail web service. All significant DEGs were sorted according to their fold change and used as input for GeneTrail with default parameters. An adjusted P value cutoff of 0.05 was applied to select the most significant enriched/depleted transcription factor complexes, regulating these DEGs. Seven depleted transcription factor complexes (q < 0.05) were identified in shRad21-LSK cells. **(D and E)** 5,000 GFP^+^LSK cells were isolated from the recipient mice of *Rad21* knockdown or control LSK cells at 4 mo after transplantation and cultured for 3 d. The percentage of CD11b^+^ cells in the cultures was determined by FACS. **(D)** Representative FACS plots showing percentages of CD11b^+^ cells in the indicated groups. **(E)** The histogram shows the percentage of CD11b^+^ cells in the LSK cell cultures in the indicated groups (in total, *n* = 8 mice per group were analyzed in *n* = 2 independent experiments). Statistical significance was assessed with two-way ANOVA followed by Tukey’s multiple comparison test on logit-transformed data. **(F)** LSK cells from 16-mo-old *NF-κB1*^−/−^ or wild-type mice were infected with viruses carrying shRNAs against *Rad21* or luciferase and cultured with or without LPS/Pam3 for 3 d. The graphs depict the increase in differentiation of LSK cells into myeloid cells (CD11b^+^) in LPS/Pam3-treated cultures relative to PBS-treated cultures of LSK cells from wild-type mice (left) and LSK cells from *NF-κB1*^−/−^ mice (right) that were infected with the indicated shRNAs. The dots represent individual mice (in total, *n* = 6 wild-type mice and *n* = 4 *NF-κB1*^−/−^ mice were analyzed in *n* = 2 independent experiments). Statistical significance was assessed with paired *t* test after log transformation. All data represent mean ± SD; *, P < 0.05; ****, P < 0.0001; ns, not significant.

To use an unbiased approach to identify pathways that may contribute to enhanced selection of *Rad21*/cohesin-deficient HSPCs in the context of stressed hematopoiesis, RNA sequencing (RNA-seq) was performed on *Rad21* shRNA versus control shRNA-infected HSPCs (LSK cells) that were reisolated after one round of repopulation stress in vivo or 3 d after activation in culture. Kyoto Encyclopedia of Genes and Genomes (KEGG) pathway analysis on differentially expressed genes (DEGs) revealed a significant inhibition of NF-κB–dependent signaling in response to *Rad21* knockdown (Fig. 4, A and B; and Fig. S2 A). Culture-activated LSK cells compared with freshly, reisolated LSK cells from long-term engrafted recipient mice exhibited a higher induction of NF-κB/inflammation signaling and thus a stronger rescue of NF-κB–dependent signaling by *Rad21* knockdown (Fig. 4, A and B; and Fig. S2 A). A possible explanation for these results could be the fact that repopulation stress was already alleviated in vivo, as most HSCs had reentered quiescence 4 mo after transplantation. In contrast, the culture-induced activation of LSK cells is known to evoke an acute activation stress likely being associated with a higher induction of NF-κB signaling (Glimm et al., 2000; Pietras et al., 2011; Magnusson et al., 2013). Importantly, *Rad21* knockdown reduced the expression of the vast majority of NF-κB target genes in both settings (Fig. 4 B and Fig. S2 A). GeneTrail web service (Stöckel et al., 2016) was used on all DEGs in culture-activated *Rad21*-deficient versus control LSK cells to determine transcription factor complexes that are predicted to contribute to the regulation of these genes. Of note, NF-κB complexes accounted for six out of the top seven (q < 0.05) predicted regulator complexes (Fig. 4 C).

To functionally test the role of *Rad21* in mediating inflammation/NF-κB signaling, as well as its consequences on HSC self-renewal and differentiation, LSK cells were isolated from stably engrafted mice, 4 mo after transplantation with lentivirally transduced HSPCs carrying shRNAs against *Rad21* or a control shRNA. Freshly isolated LSK cells were exposed for short-term ex vivo culture to LPS/Pam3 to stimulate NF-κB signaling. Recent studies revealed that LSK cells are highly responsive to activate NF-κB signaling in response to LPS/Pam3 (Zhao et al., 2014). LPS/Pam3 treatment led to robust induction of myeloid differentiation of LSK cells, which was not seen in untreated cultures (Fig. 4, D and E). Of note, *Rad21* knockdown strongly abrogated LPS/Pam3-induced differentiation of freshly isolated LSK cells cultured for 3 d (Fig. 4, D and E). Similar results were seen after knockdown of other components of the cohesin complex (*SA1*, *SA2*, and *SMC3*) confirming that cohesin inhibition suppresses LPS/Pam3-induced differentiation of LSK cells into myeloid cells (Fig. S2 B).

Reduction of LPS/Pam3-induced differentiation in *Rad21*-deficient LSK cells was associated with impaired activation of NF-κB targets as compared with control cells (Fig. S2 C). After a 4-d culture, LSK cell differentiation was also induced in the absence of LPS/Pam3 stimulation, and this culture-induced differentiation was impaired by *Rad21* knockdown (Fig. S2 D). Stimulation of these cultures with IL-6 (a prominent downstream target of NF-κB) led to induction of myeloid differentiation in both shRNA-*Rad21*– and shRNA-*luci*–infected LSK cells, but the overall rate of differentiation remained significantly suppressed in *Rad21* knockdown cells (Fig. S2 D). These data indicated that *Rad21* mediates inflammation and culture-induced differentiation, which is driven by the *Rad21*-dependent induction of different NF-κB target genes including *Il6*. Stimulation with IL-6 retained the ability to induce differentiation of *Rad21*-deficient cells, suggesting that NF-κB targets can induce differentiation independent of *Rad21.* According to this result, the overall reduction of differentiation in *Rad21* knockdown cells likely involves a combined effect of the suppressed activation of different NF-κB target genes including a variety of secreted cytokines. The full rescue of the differentiation defects in *Rad21*-depleted HSPCs in secondary transplantations at the 4-wk time point (known to be associated with strong induction of various cytokines) supports this interpretation (Fig. 3, E and F).

Aiming to determine to what extent the inhibitory effect of *Rad21*/cohesin knockdown on LPS/Pam3-induced differentiation was functionally dependent on NF-κB signaling, HSPCs from NF-κB/*p50* knockout (*NF-κB1*^−/−^), and wild-type mice (*NF-κB1*^+/+^) were infected with shRNAs against *Rad21* or a control shRNA (shLuci) followed by LPS/Pam3 or PBS treatment. The *p50* knockout mouse represents a suitable model, as *p50* was identified to be the main mediator of NF-κB signaling–induced hematopoietic failure of Iκ-Bα–deficient mice (Beg et al., 1995). p50-mediated NF-κB signaling involves the formation of homodimer (p50:p50) and heterodimers (p50:p65) with p65, which was up-regulated in LSK cells from young versus old mice (Fig. 1 A). As seen above, the knockdown of *Rad21* strongly inhibited LPS/Pam3-induced differentiation of wild-type HSPCs (Fig. 4 F, left). The overall level of LPS/Pam3-induced differentiation was reduced in *NF-κB1*^−/−^ HSPCs compared with *NF-κB1*^+/+^ HSPCs. Importantly, *Rad21*/cohesin depletion had no inhibitory effect on LPS/Pam3-induced differentiation in the *p50* knockout background, indicating that inhibition of inflammation-induced differentiation of HSPCs by cohesin suppression was dependent on NF-κB signaling (Fig. 4 F, right).

**Figure 5.**
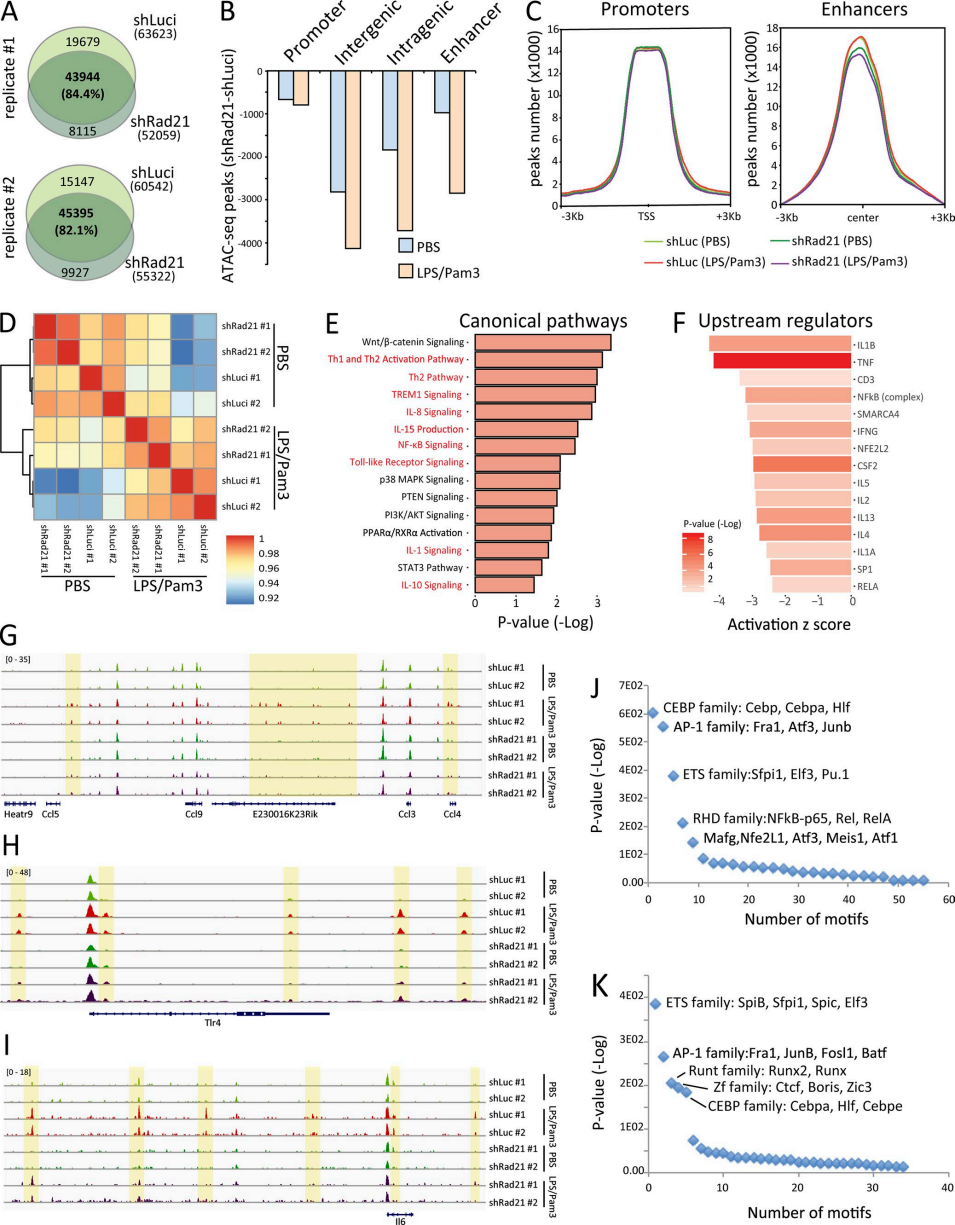
***Rad21* deficiency leads to changes in chromatin accessibility in LSK cells in response to inflammation. (A–K)** LSK cells from 24-mo-old mice were infected with an shRNA against Rad21 or a control shRNA (shLuci) and cultured for 2 d followed by an exposure to LPS/Pam3 (LPS: 200 ng/ml; Pam3: 1 μg/ml) or PBS control for 14 h. At that time point, LSK cells were subjected to ATAC-seq. **(A)** Venn diagrams showing the number of ATAC-seq peaks (P value cutoff = 1e-3) in PBS-treated LSK cells. There was a reduction in the overall number of peaks in LSK cells that were infected with an shRNA against *Rad21* versus control shRNA (shLuci)–infected LSK cells. **(B)** The histogram depicts the reduction in ATAC-seq peaks in the indicated genomic regions of shRad21 versus shLuci-infected LSK cells that were treated with PBS or LPS/Pam3. For the calculation, only common peaks found in both of the two replicates have been used. **(C)** Distribution of the ATAC-seq peaks on promoters and enhancers (±3 kb) in LSK cells infected with the indicated shRNAs. **(D)** Hierarchical clustering of the Pearson’s correlation of the ATAC-seq reads in all the samples analyzed. **(E)** Signaling pathway enrichment (x axis) of the genes associated with peaks depleted in shRad21-LPS/Pam3 compared with shLuci-LPS/Pam3–treated cells. **(F)** Upstream regulators associated with the genes depicted in E. X axis shows the activation Z-score of the target genes, and color code shows enrichment P value (−log10). **(G–I)** Genomic views of the *Ccl* gene cluster (G), *Tlr4* (H), and *Il6* (I). The yellow highlight marks the genomic regions where ATAC-seq identified increases in chromatin accessibility in response to LPS/Pam3 treatment, but diminished increases in chromatin accessibility in *Rad21* knockdown versus control shRNA infected cells. **(J and K)** Motif discovery results on the peaks enriched in shLuci-LPS/Pam3 compared with shLuci-PBS–treated cells (J) or depleted in shRad21-LPS/Pam3 compared with shLuci-LPS/Pam3–treated cells (K).

Together, our results indicated that *Rad21*/cohesin is a critical component mediating stress-induced NF-κB/inflammatory signals in transplanted or cultured HSCs and HSPCs. Cohesin has a crucial role in assisting the formation and stabilization of higher-order chromatin structures, such as topology-associated domains (TADs) and DNA loops (Rao et al., 2017; Schwarzer et al., 2017) that were shown to be important for inducible gene expression in macrophages involving the regulation of chromatin accessibility for intermediate transcription factors in stress signaling pathways (Cuartero et al., 2018). However, previous studies did not reveal a prominent role of cohesin in mediating NF-κB signaling, a key mediator of stress and inflammatory responses in various organ systems. To assess for a possible involvement of cohesin-mediated chromatin changes in controlling NF-κB target genes expression in HSPCs in stressed hematopoiesis, freshly isolated HSPCs (LSK cells) from 24-mo-old mice were infected with an shRNA against *Rad21* or a control shRNA (shLuci) and cultured for 2 d, followed by an exposure to LPS/Pam3 or PBS control for 14 h. LSK cells were harvested and subjected to assay for transposase accessible chromatin with high-throughput sequencing (ATAC-seq) analysis to determine changes in chromatin accessibility in HSPCs in response to LPS/Pam3-induced NF-κB signaling and the influence of *Rad21* knockdown on the induction of NF-κB–mediated changes in chromatin accessibility (Fig. 5). ATAC-seq of two biological replicates per condition revealed an overall reduction in chromatin accessibility in *Rad21*-deficient LSK cells versus control cells exposed to PBS (Fig. 5 A). This decrease in chromatin accessibility in *Rad21* knockdown LSK cells affected intergenic and intragenic regions more strongly than promoter regions, and the decrease was aggravated in response to LPS/Pam3 treatment (Fig. 5 B). Of note, previously identified enhancer regions in hematopoietic cells (Pundhir et al., 2018) showed reduced chromatin accessibility in *Rad21*-deficient versus control LSK cells irrespective of the exposure to LPS/Pam3 or control treatment (Fig. 5 C). Only a minor fraction (∼10%) of LPS/Pam3-induced nonpromoter peaks regions overlapped with previously annotated enhancers (Pundhir et al., 2018), suggesting that LPS/Pam3-induction may activate specific enhancers.

Pearson’s correlation analysis on chromatin accessibility peaks revealed a separate clustering of PBS versus LPS/Pam3-treated LSK cells (Fig. 5 D). However, the knockdown of *Rad21* made LPS/Pam3-treated LSK cells more similar to PBS-treated cultures (Fig. 5 D), indicating that *Rad21* knockdown diminishes LPS/Pam3-induced changes in chromatin accessibility in LSK cells. Pathway enrichment analysis of genes that were located in the vicinity of LPS/Pam3-induced chromatin accessibility peaks that were reduced by *Rad21* knockdown revealed a strong enrichment of NF-κB–related pathways (Fig. 5 E). When analyzing the upstream regulators of these genes, we could identify NF-κB as one of the main regulators (Fig. 5 F). Zooming into example regions revealed LPS/Pam3-induced increases in chromatin accessibility in inter-/intragenic regions of various genes and gene clusters that are regulated by NF-κB and that exhibited a reduced responsiveness to open the chromatin in *Rad21*-deficient versus control LSK cells (Fig. 5, G–I). Transcription factor–binding sites that were directly located in regions of LPS/Pam3-induced chromatin accessibility in shLuci-infected LSK cells showed a strong enrichment for NF-κB transcription factor–binding sites (Fig. 5 J). As expected, depleted regions of LPS/Pam3-induced chromatin accessibility in *Rad21*-deficient cells were enriched for CCCTC-binding factor (*CTCF*, Fig. 5 K), a major cofactor of *Rad21*-dependent formation of higher order chromatin structures (Parelho et al., 2008). Of note, NF-κB–binding sites were not enriched within *Rad21*-dependent regions of inflammation-induced chromatin accessibility (Fig. 5 K). Together, these experiments show that *Rad21*-dependent changes in chromatin accessibility have a major impact on the induction of NF-κB signaling in response to inflammation stimuli in HSPCs. The data suggest that *Rad21*-dependent changes in chromatin accessibility in response to inflammation mediate enhancer functionality of cotranscription factor complexes that are required for the transcriptional activation of NF-κB–dependent target genes. In line with this interpretation, the most strongly enriched transcription factors in *Rad21*-dependent regions of increased chromatin accessibility in response to inflammation included ETS, AP-1, and Runt family members (Fig. 5 K), which are known cofactors for NF-κB signal induction (Thanos and Maniatis, 1995; Luo et al., 2016).

### Aging-associated changes in the effects of cohesin-mediated NF-κB-signaling on HSPC expansion in response to inflammation stimulation

The above data indicated that HSPCs from old compared with young mice exhibit an increased ground-stage activity of NF-κB signaling (Fig. 1) and an increased sensitivity to undergo differentiation and loss of self-renewal in response to induction of NF-κB signaling in unperturbed, endogenous hematopoiesis (Fig. 2). Moreover, *Rad21*/cohesin was identified as a mediator of NF-κB signaling in murine HSPCs by regulating chromatin accessibility in regulatory inter-/intragenic and enhancer regions in response to inflammatory stimuli (Fig. 4 and Fig. 5). To test whether aging would change the fate of HSPCs in response to inflammation-induced NF-κB stimulation and to assess whether *Rad21*/cohesin is potentially involved in this process, freshly isolated LSK cells from young (2 mo old) and old (24 mo old) mice were infected with a shRNA against *Rad21* or a control shRNA. 2 d after infection, LSK cells were exposed to LPS/Pam3 or control (PBS) treatment and cultured for another 3 d. LPS/Pam3 treatment induced myeloid differentiation in HSPC cultures from both young and old mice (Fig. S3, A and C). However, an age-dependent difference was seen in the expansion potential of HSPCs in response to inflammation (Fig. S3, B and D). Specifically, LPS/Pam3 treatment compared with PBS treatment strongly inhibited the expansion potential of old HSPCs (Fig. S3 D) but increased the expansion of young HSPCs (Fig. S3 B). Of note, *Rad21* knockdown completely rescued the inhibition of the expansion potential of old HSPCs exposed to LPS/Pam3 treatment and reverted the enhancement of expansion of LPS/Pam3-treated young HSPCs (Fig. S3, B and D).

Similar results were obtained by NF-κB inhibition (Fig. S3, E–H). In brief, freshly isolated LSK cells from young (2 mo old) and old (24 mo old) mice were placed into culture and exposed to LPS/Pam3 to induce NF-κB signaling. Cultures were analyzed 3 d later. As seen in the experiment on virally transduced cells, LPS/Pam3 treatment induced myeloid differentiation (CD11b^+^) in HSPC cultures from both young and old mice (Fig. S3, E and G), but exerted age-dependent effects on HSPC expansion resulting in the inhibition of the expansion potential of HSPCs from aged mice (Fig. S3 H), whereas the expansion of HSPCs from young mice was increased (Fig. S3 F). Treatment of HSPC cultures with BMS-345541 (BMS), a highly selective inhibitor of NF-κB–dependent transcription (Burke et al., 2003), reverted impairments in the expansion potential of old HSPCs as well as increases in the expansion potential of young HSPCs in the context of inflammation stimulation (Fig. 3, F and H). The impaired expansion potential of old HSPCs in response to LPS/Pam3 stimulation did not associate with an increased induction of apoptosis as compared with cultures from young HSPCs (Fig. S3 I), which was also confirmed at an earlier time point of inflammation induction in vivo (24 h after LPS injection, see below; Fig. S3 J).

Together, these data revealed an aging-dependent response of HSPCs to the induction of inflammation/NF-κB signaling in culture, resulting in impaired expansion potential of HSPCs from old versus young mice. These divergent responses to inflammation/NF-κB activation are *Rad21* dependent and can be rescued by *Rad21* knockdown.

### Resolution failure of inflammatory signals impairs expansion and induces differentiation of HSCs in response to inflammation stimulation in aging mice

**Figure 6.**
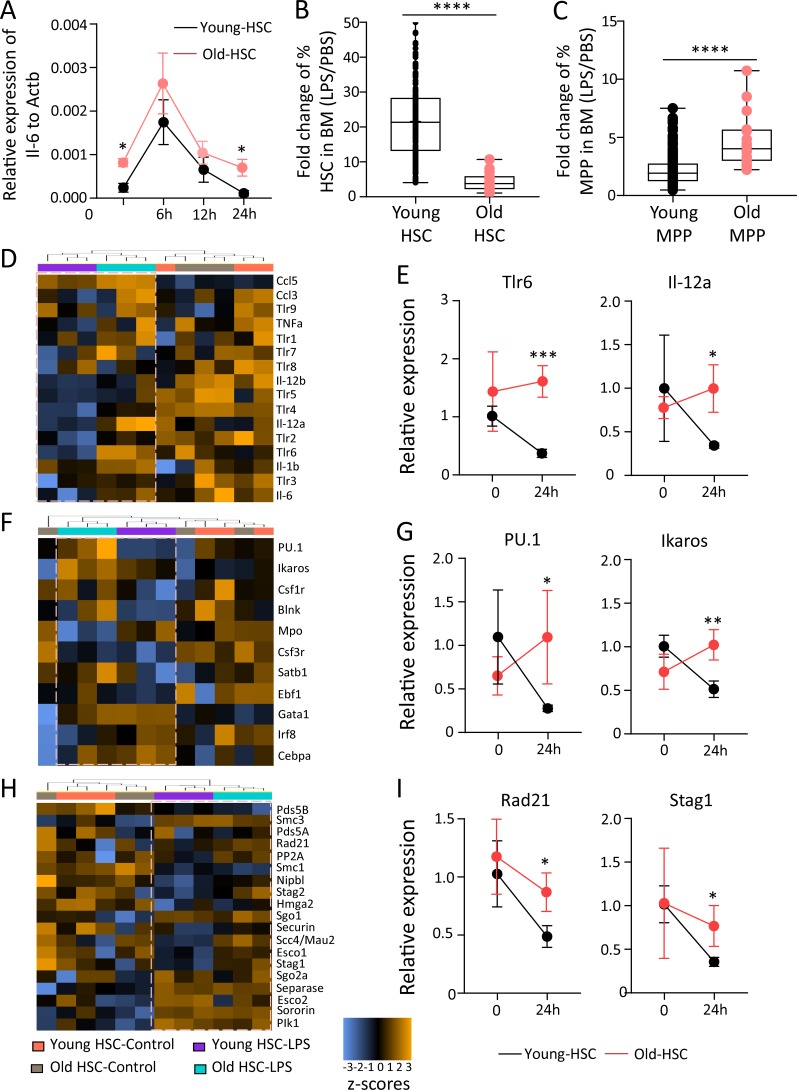
**Age-dependent impairment in expansion potential of HSCs in response to LPS exposure associates with a failure to down-regulate inflammation signaling and cohesin expression in the resolution phase of inflammation. (A)** HSCs (CD150^+^CD34^−^LSK) were isolated from young (2–3 mo old) and old (24 mo old) wild-type mice and treated with PBS or LPS (200 ng/ml) for 2 h. mRNA expression of *Il6* relative to *Actb* was analyzed at the indicated time points after LPS stimulation (*n* = 3–4 mice per group). Statistical significance was assessed with Welch’s *t* test after log transformation. **(B–I)** In vivo LPS stimulation was performed in young (2–3 mo old) and old (24 mo old) wild-type mice at a dose of 1.5 mg/kg body weight. Mice were sacrificed at 24 h after LPS injection. **(B and C)** The charts depict the fold change in the percentages of HSCs (B) and MPPs (C) in BM of LPS-stimulated mice relative to control (PBS injected) mice of the indicated age groups. Multiple pairwise comparisons of fold changes of HSCs (CD150^+^CD41^−^CD34^−^LSK; B) and MPPs (CD34^+^ LSK; C) were performed. Wilcoxon test was used to test difference of all pairwise ratios for each cell type (in total, *n* = 16 young mice and *n* = 4 old mice were analyzed in four independent experiments). **(D–I)** NanoString analysis was performed on HSCs from young (2–3 mo old) and old (24 mo old) mice, 24 h after LPS injection along with controls. The heat maps show the clustering of the indicated groups according to changes in the mRNA expression of inflammatory genes (D), differentiation factors (F), and cohesin genes and cohesin regulator genes (H). **(E, G, and I)** Histograms show the relative mRNA expression of representative genes encoding for cytokines (E), differentiation inducing factors (G), or cohesin components (I). The depicted representative examples were selected from the NanoString analysis in D, F, and H. The data are shown as linear fold change relative to the nontreated young control (*n* = 12 young mice in three pools of 4 mice per pool, and *n* = 3 old mice as individual samples). 12 housekeeping genes were used for the normalization of the expression value. Statistical significance was assessed with Welch’s *t* test after log transformation. Data represent mean ± SD; *, P < 0.05; **, P < 0.01; ***, P < 0.001; ****, P < 0.0001.

To determine signaling pathways that contribute to increased sensitivity of aged HSCs to lose expansion potential in response to inflammatory stimuli, we performed a time course experiment on freshly isolated HSCs, which were exposed to low-dose LPS for 2 h, followed by culture in LPS-free medium for 6, 12, and 24 h. This experiment reconfirmed an increased ground-stage activity of NF-κB transcription (as measured by *Il6* mRNA expression) in freshly isolated HSCs from old (24 mo old) compared with young (2–3 mo old) mice (Fig. 6 A). LPS stimulation did not result in significant changes in peak activation of *Il6* transcription. However, in the resolution phase after LPS stimulus, HSCs from old mice failed to down-regulate *Il6* levels below the ground-stage expression (Fig. 6 A).

To determine whether aging would change the cell fate of HSPCs in response to inflammation stimuli in vivo and whether such changes may be related to age-dependent changes in the resolution phase of inflammatory responses, young (2–3 mo old) and old (24 mo old) mice were injected with a single dose of LPS. Inflammatory responses and *Rad21*/cohesin expression were analyzed in freshly isolated HSPCs 24 h after LPS injection (1.5 mg/kg body weight). LPS-induced stimulation of inflammation resulted in a strong expansion of phenotypically defined HSCs (CD150^+^CD41^−^CD34^−^LSK) in both young and old mice (Fig. 6 B and Fig. S4 A). However, this expansion was diminished in BM of old mice, which exhibited instead a stronger increase in the number of MPPs (CD34^+^LSK) in response to LPS exposure compared with young mice (Fig. 6, B and C; and Fig. S4, A and B).

The increase in total number of phenotypically defined HSCs per million BM cells was to some extent due to a decrease in total BM cells in LPS-injected versus PBS-injected mice, likely due to the known mobilization of BM cells into the periphery in response to inflammation (Fig. S4 C). However, the magnitude of the decrease of BM cells in response to LPS injection was similar in young and old mice indicating that it did not influence age-dependent changes in HSC percentages in response to LPS. Cell cycle analysis revealed increased proliferation of both HSCs and MPPs in LPS-injected versus PBS-injected mice of both age groups, but the percentage of HSCs and MPPs in S/G2/M stages in response to LPS did not show a significant difference in young versus old LPS-injected mice (Fig. S4, D and E; P = 0.7660 for HSCs and P = 0.7764 MPPs). Similarly, the rate of apoptosis did not show significant age-related changes among the groups (Fig. S3 J). However, changes in surface marker expression could influence the quantification of HSCs and MPPs in response to LPS-induced inflammation, as such changes have been described in response to interferon stimulation in vivo (Essers et al., 2009). Despite these considerations, the acquired data suggested that phenotypically defined HSCs exhibit a diminished expansion potential and an increased induction of differentiation toward MPPs in old compared with young mice in response to an acute inflammatory stimulus. This interpretation also stands in agreement with our above analysis of old versus young NF-κB reporter mice showing an age-dependent increase in differentiation and a reduced expansion potential of HSCs after endogenous activation of NF-κB signaling in steady-state hematopoiesis of non-LPS–treated mice (Fig. 2).

**Figure 7.**
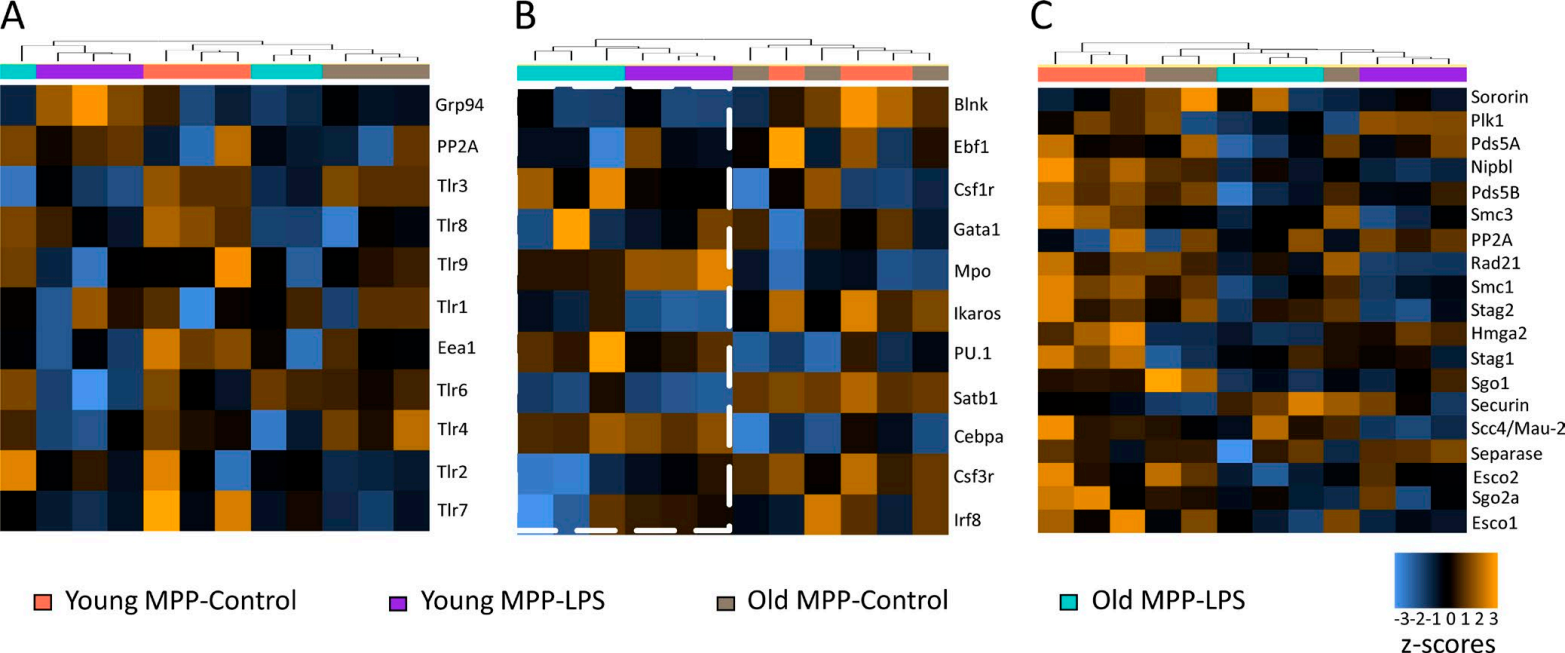
**mRNA expression signatures of MPPs upon in vivo LPS stimulation. (A–C)** NanoString analysis was performed on MPPs (CD34^+^LSK) from young (2 mo old) and old (24 mo old) mice, 24 h after injection of LPS (1.5 mg/kg body weight) or control, PBS injection (*n* = 12 young mice in three pools of 4 mice per pool, and *n* = 3 old mice as individual samples; the same experiment as depicted in Fig. 6, D–I). The heat maps show the clustering of the indicated groups according to changes in the mRNA expression of inflammatory genes (A), differentiation factors (B), and cohesin genes and cohesin regulator genes (C).

To determine whether age-dependent differences in the resolution of inflammation/differentiation signals may be involved in age-related changes how HSCs respond to inflammation stimulation in vivo, a NanoString mRNA expression analysis was conducted on freshly isolated, highly purified HSCs (CD150^+^CD41^−^CD34^−^LSK) and MPPs (CD34^+^LSK) from the same mice (24 h after LPS injection). Pathway directed analysis confirmed that HSCs from young LPS-injected mice had already entered a resolution phase of inflammation as indicated by a reduction in TLR/cytokine signaling (Fig. 6, D and E) and differentiation-inducing signals (Fig. 6, F and G) in LPS-injected compared with control animals. Control animals exhibited an increased expression of several NF-κB target genes in HSCs from old versus young mice (such as *Tlr5*, *Tlr4*, *Il12b*, and *Il6*) reconfirming an increased ground stage of NF-κB activity as seen before (Fig. 1 and Fig. 6 A). Interestingly, HSCs from aged, LPS-injected mice showed an attenuated resolution response characterized by a failure to down-regulate inflammation- and differentiation-inducing pathways in HSCs from LPS-injected mice versus control mice (Fig. 6, D–G). Of note, these aging-associated changes in the resolution phase after LPS injection associated with a down-regulation of several components of the cohesin complex (*Rad21*, *Stag2*, and *Stag1*) and factors that load/stabilize cohesin on the chromatin (*Nipbl* and *Scc4/Mau-2/Securin*) in HSCs from LPS-injected young mice, but a failure in cohesin down-regulation in HSCs from LPS-injected old mice in comparison to age-matched control animals (Fig. 6, H and I). In contrast to the data on HSCs, age-dependent changes in resolution phase and cohesin down-regulation after LPS injection were not evident in MPPs (Fig. 7, A–C), suggesting that the age-dependent failure in resolution of inflammation is more prominent at the level of HSCs.

### Selection of *Rad21*-deficient HSCs with reduced and myeloid-biased (My-biased) differentiation capacity increases during aging

Together, the above data suggested that an aging-associated failure in down-regulation of *Rad21*/cohesin and inflammation/differentiation-inducing signals in HSCs contributes to the increased sensitivity of HSCs from old versus young mice to undergo inflammation-induced loss of self-renewal/expansion potential and induction of differentiation. The endogenous activation of NF-κB in non-LPS injected NF-κB reporter mice indicated that this pathway is active during steady-state hematopoiesis in the absence of acute inflammatory stimuli, and of note, aging changes the cell fate of NF-κB–activated HSCs to undergo differentiation and loss of self-renewal in unperturbed hematopoiesis (Fig. 2). Based on these lines of evidence, we postulated that *Rad21*/cohesin-deficient HSCs could be selected during aging.

**Figure 8.**
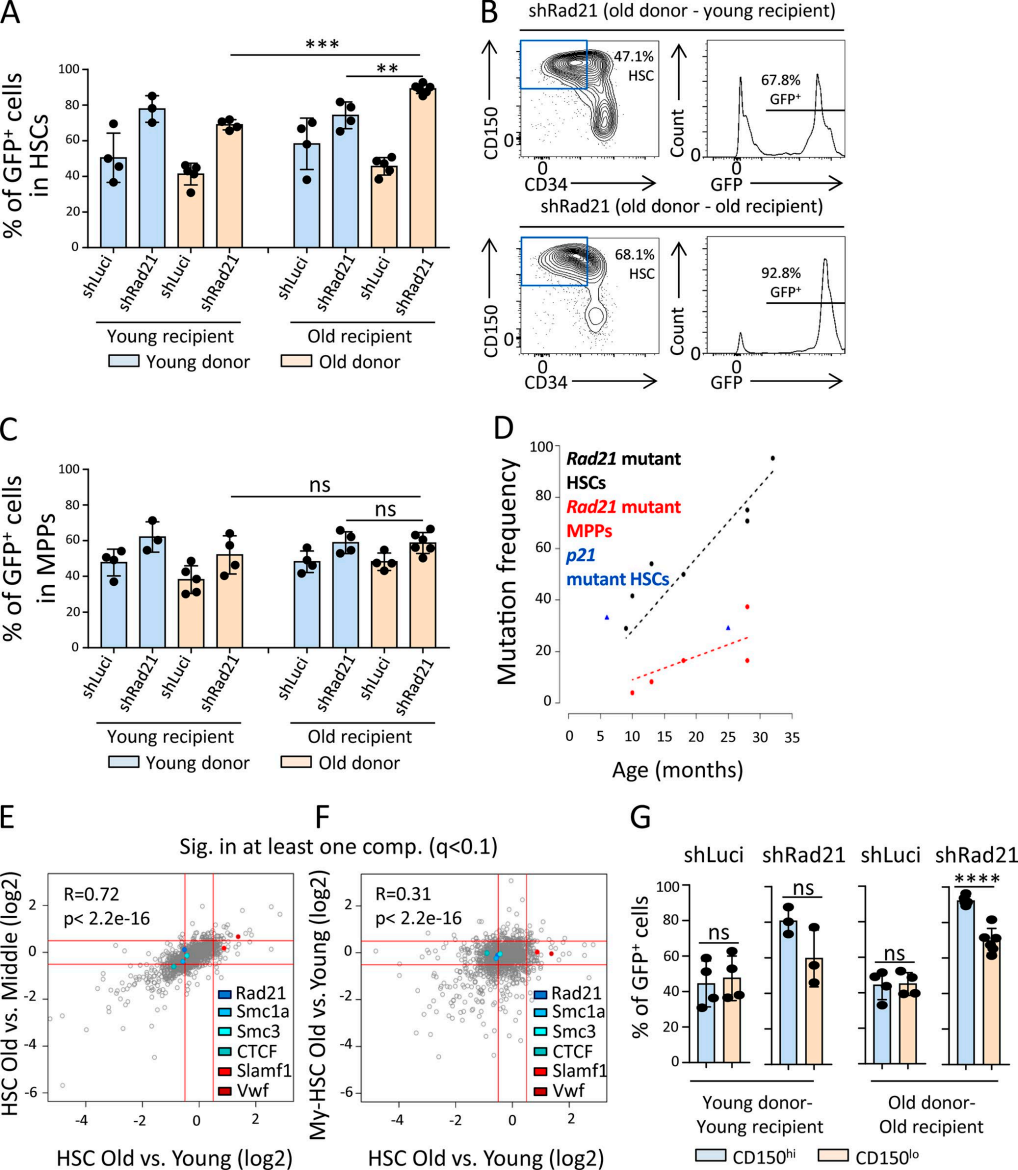
**HSCs with disrupted cohesin expression are selected during aging. (A–C)** LSK cells from young (2 mo ­old) or old (24 mo old) donor mice were infected with an shRNA against *Rad21* (shRad21) or a control shRNA (shLuci) and transplanted into young (2 mo old) or old (15 mo old) recipient mice along with noninfected LSK cells from the same cultures. The initial transduction efficiencies were 69.6% for shLuci-young-LSK, 72.2% for shRad21-young-LSK, 64.9% for shLuci-old-LSK, and 69.5% for shRad21-old-LSK. The percentage of GFP^+^ cells was determined 9 mo after transplantation in HSCs and MPPs of the recipient mice. **(A)** The histogram depicts the percentages of GFP^+^ cells in HSCs (CD150^+^CD34^−^LSK) in the indicated groups. **(B)** Representative FACS plots showing (left) the percentage of HSCs in LSK cells and (right) the percentage of GFP^+^ cells in the HSC population in the indicated groups of recipient mice transplanted with targeted LSK cells from old donors. **(C)** The histogram depicts the percentages of GFP^+^ cells in MPPs (CD34^+^LSK) in the indicated groups. **(A and C)** In total, *n* = 3–6 recipient mice per group in *n* = 3 independent experiments. Statistical significance was assessed with two-way ANOVA followed by Tukey’s multiple comparison test on logit-transformed data. The dots represent individual mice. **(D)** Heterozygous floxed *Rad21* mice and heterozygous floxed *p21* mice that also carried an Mx-Cre transgene (*Rad21*^fl/+^, Mx-Cre^+^ or *p21* ^fl/+^, Mx-Cre^+^) were analyzed at the indicated age without pIpC-mediated Cre induction. The chart depicts the rates of heterozygous gene deletion (y axis) in HSCs and MPPs from the mice of the indicated age (x axis) as determined by genotyping colonies formed from single cells. *n* = 24–50 colonies per cell type per mouse, *n* = 2–7 mice per genotype in total in *n* = 3 independent experiments. Regression analysis was performed using the standard least squares estimator to determine the association of age with mutation frequency of *Rad21* mutant HSC/MPP. Z-test was used to test difference in slope of regression lines between HSC and MPP populations tested (P < 2e-16). **(E)** Scatter plot comparing proteome changes in (x axis) CD150^+^ HSCs (including CD150 high and low = My- and Ly-biased HSCs) from old mice (24 mo old) versus young mice (2–4 mo old) and in (y axis) CD150^+^ HSCs from old mice (24 mo old) versus middle age mice (10–14 mo old). Only protein groups significantly affected in at least one of the two comparisons (q < 0.1) are shown (1,161 protein groups). *n* = 3–4 pools per group of *n* = 3–12 mice per pool. **(F)** Scatter plot comparing proteome changes in (y axis) purified CD150^hi^ HSCs (My-biased HSCs) from old mice (30 mo old) versus young mice (6–8 mo old) and in (x axis) CD150^+^ HSCs (including CD150^hi^ and CD150^lo^ = My- and Ly-biased HSCs) from old mice (24 mo old) versus young mice (2–4 mo old). Only protein groups significantly affected in at least one of the two comparisons (q < 0.1) are shown (1,468 protein groups). *n* = 3–4 pools per group of *n* = 3–12 mice per pool. **(G)** The histogram shows the percentages of GFP^+^ cells in CD150^hi^ (My-biased) and CD150^lo^ (Ly biased) HSCs in the indicated groups (data derived from the same experiment as depicted in Fig. 8, A–C). The dots indicate individual mice. Statistical significance was assessed with Welch’s *t* test on logit-transformed data. Data represent mean ± SD; **, P < 0.01; ***, P < 0.001; ****, P < 0.0001; ns, not significant.

To directly test whether aging-associated changes in the cell fate of HSCs in response to NF-κB activation would enhance the selection of *Rad21*-deficient HSCs during in vivo aging, LSK cells were isolated from young (2 mo old) and old (24 mo old) donor mice, infected with a *Rad21*-shRNA or a control shRNA, and transplanted along with noninfected cells into young (2 mo old) or old (15 mo old) recipient mice. 9 mo after transplantation, the chimerism of shRNA-infected (GFP^+^) cells was analyzed in the HSC population (CD150^+^CD34^−^LSK) of the recipient mice. Positive selection of *Rad21*-deficient HSCs compared with control shRNA HSCs was evident in all groups of recipient mice (Fig. 8, A and B). Interestingly, a further increase in the selection of *Rad21-*deficient HSCs occurred when HSCs from old donors were transplanted into old recipients, whereas each parameter alone (an advanced age of the donor HSPCs or an advanced age of the recipient mice) was not sufficient to lead to an enhancement in the selection of *Rad21*-deficient HSCs (Fig. 8, A and B). Aging-associated increases in the selection of *Rad21-*deficient cells were not seen at the level of MPPs from the same animals (Fig. 8 C).

These results indicated that aging-related HSC-intrinsic processes as well as HSC-extrinsic processes cooperate in enhancing the selection of *Rad21*-deficient HSCs but not MPPs after transplantation. To characterize the selection of *Rad21* mutant HSCs in unperturbed hematopoiesis during aging, a cohort of seven *Rad21*^fl/+^Mx-Cre^+^ mice was analyzed. The Mx-Cre system allows for inducible deletion of knockout alleles that are flanked by loxP sites. The Cre driver can be induced by interferon activation through injection of double-stranded RNA polyinosinic-polycytidylic acid (pIpC). However, the Cre driver has a low background activity, resulting in a low percentage of deleted alleles in the absence of pIpC injection ranging from 1 to 30% (Kühn et al., 1995; Velasco-Hernandez et al., 2016). We reasoned that this model could be used to determine kinetics of positive selection of heterozygous mutant *Rad21* (*Rad21^Δ/+^*) HSCs and MPPs during aging. Interestingly, a strong aging-associated increase in the percentage of mutant HSCs was seen in these mice reaching up to 70.8–95.2% in three 28–33-mo-old mice (Fig. 8 D). As a control, we determined the rate of p21 mutant HSCs in one 6-mo-old and one 25-mo-old p21^fl/+^Mx-Cre mouse. Homozygous deletion of p21 was found to increase self-renewal in mice of a mixed genetic background (Cheng et al., 2000), but such effects were not seen in mice on a C57BL/6J background (van Os et al., 2007). The percentage of mutant HSCs reached to 29.1% and 33.3% in these two control mice (Fig. 8 D). While the number of mice was too low to make conclusion on mutation selection kinetics in unperturbed hematopoiesis of aging mice of these different genotypes, we wished to determine whether *Rad21* mutant HSCs would change the differentiation potential of HSCs. To this end, the frequency of *Rad21* deletion was determined for five of the seven investigated mice also at the level of MPPs. Our above data indicated that *Rad21* knockdown increases self-renewal but impairs the differentiation capacity of HSCs (Fig. 3). Interestingly, the in vivo accumulation of *Rad21* mutations in the five investigated mice was much lower in MPPs than in the HSC compartment of the same mice (Fig. 8 D). Also, the slope of age-related increases in mutations was significantly lower in MPPs compared with HSCs from the same mice (Fig. 8 D; P < 10^−5^), suggesting that *Rad21* mutations are stronger selected at HSC level compared with MPP level.

During aging, the population of My-biased HSCs strongly expands, whereas the population of lymphoid-biased (Ly-biased) HSCs does not increase (Wang et al., 2016). This leads to a dominance of My-biased HSCs and My-biased differentiation in the aging hematopoietic system (Cho et al., 2008; Beerman et al., 2010; Dykstra et al., 2011). My-biased HSCs exhibit an increased expression of CD150 and von Willebrand Factor (VWF), and the population of HSCs expressing these markers increases during aging (Beerman et al., 2010; Morita et al., 2010; Pinho et al., 2018). To analyze whether increases in the selection of HSCs with reduced protein expression of *Rad21/*cohesin would occur during aging and may cosegregate with the selection of HSC subpopulations, global proteome analysis was performed on freshly isolated HSCs from young (2–4 mo old), middle age (10–14 mo old), and old (24 mo old) mice. HSCs were isolated as CD150^+^CD34^−^LSK cells containing both My-biased (CD150-high-positive: CD150^hi^) and Ly-biased (CD150-low-positive: CD150^lo^) HSCs. As expected, when analyzing the entire pool of My-biased and Ly-biased HSCs, significant increases in the expression of marker proteins of My-biased HSCs (CD150 and VWF) were detected during aging (q < 0.1; Fig. 8 E). This aging-associated increase in marker proteins of My-biased HSCs (CD150 and VWF) was more pronounced when comparing HSCs from old versus young mice as compared with HSCs from old versus middle-aged mice (Fig. 8 E). This observation stands in agreement with the age-dependent increase in My-biased HSCs, which becomes prominently detectable already at mid age (Tang et al., 2016). Interestingly, these aging-associated increases in the expression of My-biased HSC marker proteins were associated with a significant decrease in the expression of several components of the cohesin complex, including RAD21, SMC1A, and SMC3, as well as the binding partner for cohesin on the chromatin, CTCF, in HSCs from aged versus young mice (Fig. 8 E). However, this decrease in the expression of cohesin components was less pronounced when comparing old versus middle-aged mice (q < 0.1; Fig. 8 E). These data suggested that My-biased HSCs may express lower levels of *Rad21*/cohesin than Ly-biased HSCs, thus the selection of My-biased HSCs during aging associated with decreasing expression of *Rad21*/cohesin in the HSC pool. To further test this assumption, a second proteome experiment was conducted on purified My-biased HSCs (CD150^hi^CD34^−^LSK cells) from young mice (6–8 mo old) and old mice (30 mo old). As expected, the proteome analysis of highly purified My-biased HSCs did not show an increased expression of marker protein of My-biased HSCs (CD150 and VWF) when comparing HSCs from aged versus young mice (Fig. 8 F). Of note, the decrease in the expression of proteins of the cohesin complex, which was seen when analyzing the entire pool of My- and Ly-biased HSCs from old versus young mice (Fig. 8 F, x axis), was also no longer detectable in this highly purified population of My-biased HSCs (Fig. 8 F). Together, these data support the conclusion that the expression of proteins of the cohesin complex is reduced in My-biased HSCs versus Ly-biased HSCs and the aging-associated drift toward a My-biased HSC pool thus associates with reduction in the expression of proteins of the cohesin complex.

The above results suggested that the reduced expression of cohesin complex proteins may contribute to the selection of My-biased HSCs during aging as the reduced expression levels of cohesin would make this subpopulation of HSCs more resistant to inflammation/NF-κB–mediated impairments in HSC self-renewal/expansion. To test this hypothesis, we analyzed the selection of shRad21-infected versus shLuci-infected LSK cells from young or old donor mice upon transplantation into young or old recipient mice (same experiment as depicted in Fig. 8 A) within the subpopulations of My-biased and Ly-biased HSCs. While Luci-shRNA–infected cells did not show an aging-associated selection in My-biased versus Ly-biased HSCs, shRNA-*Rad21* infection showed an increased selection in the subpopulation of My-biased HSCs compared with Ly-biased HSCs (Fig. 8 G). These data suggested that reducing levels of *Rad21* increase the selection of My-biased HSCs more strongly than the selection of Ly-biased HSCs, supporting the hypothesis that the naturally occurring reduction in RAD21 protein expression in My-biased HSCs versus Ly-biased HSCs (Fig. 8, E and F) may contribute to the aging-associated selection of My-biased over Ly-biased HSCs.

## Discussion

The current study provides experimental evidence that the response of HSPCs to inflammation/NF-κB signaling changes during aging, which leads to an aging-associated loss of expansion/self-renewal capacity of HSPCs exposed to inflammatory stimuli. The study shows that HSCs from aged compared with young mice exhibit an increased ground-stage activity of NF-κB signaling and an elevated sensitivity to lose self-renewal and expansion capacity in response to activation of inflammation/NF-κB signaling. These aging-associated impairments in self-renewal and expansion capacity of aged HSCs are associated with failures to down-regulate inflammation/NF-κB signaling in the resolution phase of inflammation below the already increased ground-stage activity of the pathway. While NF-κB signaling can promote inflammation resolution in leukocytes (Lawrence et al., 2001), the here-described increase in ground-stage NF-κB activity and the failure to down-regulate the pathway in the resolution phase of inflammation associate with impaired self-renewal/expansion potential of HSCs in aged versus young mice in response to inflammation stimulation. Studies on young mice revealed that HSPCs are highly responsive to activate NF-κB inflammatory signals in response to LPS/TLR-induced inflammation signals, which exceeds the responsiveness of mature immune cells (Zhao et al., 2014). In that study, IL-6 was shown to represent one of the strongest induced cytokines in HSPCs in response to inflammation stimulation. Of note, IL-6 is an important activator of myeloid differentiation in HSPCs, and the up-regulation of IL-6 was identified as one of the first biomarkers of aging in humans (Franceschi and Campisi, 2014). It is conceivable that the HSPC compartment is more vulnerable than other (mature) cells of the blood system to respond to chronic increases in inflammatory signals that occur during aging. The failure to down-regulate NF-κB signaling in response to an acute inflammatory stimulus in aged HSCs below the level of already elevated ground-stage activity likely contributes to the here-identified aging-associated changes in cell fate of HSPCs to undergo differentiation and loss of self-renewal in response to acute inflammatory stimuli.

This study identifies a novel role of *Rad21*/cohesin in mediating NF-κB signaling in HSCs. Aside from its role in sister chromatid cohesion, the cohesin complex has been recognized as a regulator of gene transcription (Gullerova and Proudfoot, 2008; Yan et al., 2013). Cohesin is required for the stabilization of higher order chromatin structures, such as TADs, which almost completely disappear in response to cohesin depletion (Rao et al., 2017). However, the effects of cohesin-mediated TAD formation on gene regulation remains incompletely understood, and the removal of TADs by itself does not lead to strong changes in steady-state gene expression (Nora et al., 2017; Rao et al., 2017). The current study indicates that *Rad21*/cohesin mediates NF-κB signaling in HSPCs in response to inflammation and replication stress. Previous studies did not reveal the here identified, prominent role of *Rad21*/cohesin in mediating NF-κB signaling in HSPCs. These data indicate that *Rad21*/cohesin-mediated NF-κB signaling in HSPCs depends on the context and the presence of stress factors that activate NF-κB signals. Such factors likely were more prominent in our experiments on culture-activated or in vivo–transplanted HSPCs compared with previous studies on unperturbed hematopoiesis in young mice (Mullenders et al., 2015; Viny et al., 2015). Given that *Rad21*/cohesin deletion has little effects on gene regulations at steady-state despite its profound effects on disrupting the maintenance of topology-associated chromatin domains (Nora et al., 2017; Rao et al., 2017), it is possible that *Rad21*/cohesin is more important for inducible gene regulation, which in case of inflammation and aging involves a profound effect of *Rad21*/cohesin on mediating NF-κB signaling. In further support of our finding that cohesin can mediate NF-κB signaling in primary HSPCs, *STAG2* (*SA2*)/cohesin was reported to activate NF-κB signaling in Jurkat cells, an immortalized human T lymphocyte cell line (Lara-Pezzi et al., 2004). The current experiments reveal that *Rad21*/cohesin is required for inflammation-induced increases in chromatin accessibility in nonpromoter regions of NF-κB target genes. While NF-κB–binding motifs do not directly localize into regions of *Rad21*/cohesin-mediated increases in chromatin accessibility in response to inflammation, binding sites of several known cotranscription factors of NF-κB, such as ETS, AP1, and Runt family members (Thanos and Maniatis, 1995; Luo et al., 2016), show a strong enrichment in these regions. These findings suggest that *Rad21*/cohesin mediates inflammation/NF-κB signaling in HSPCs by inducing the enhancer activity of critical cotranscription factors of NF-κB–dependent transcription. This interpretation stands in agreement with the recently described role of *Rad21*/cohesin in mediating inducible gene expression in macrophages by activating gene expression networks through the induction of transcription factor activity and secondary gene expression responses (Cuartero et al., 2018). The current study suggests that *Rad21*-induced enhancer function of cotranscription factors has a crucial role for the induction of NF-κB–dependent inflammatory responses in HSPCs.

According to the here presented results, *Rad21*/cohesin seems to contribute to the failure of aged versus young HSCs to down-regulate inflammation/NF-κB and differentiation signals in the resolution phase after acute inflammation stimulation. This failure of aged HSCs in inflammation resolution associates with impaired down-regulation of *Rad21*/cohesin expression/stabilization. It will be of future interest to delineate the contribution of *Rad21*/cohesin to inflammatory memory and whether this may be involved in driving aging-associated phenotypes of the hematopoietic system. It has been shown that HSPCs maintain inflammatory memory, promoting an enhanced response to inflammatory insults in macrophages. This exacerbated response in the macrophages is a consequence of changes in the chromatin dynamics (Kaufmann et al., 2018). It remains to be investigated whether similar responses occur at HSC level and whether *Rad21*/cohesin is involved in these processes. The future development of methods that allow the investigation of dynamic structural/chromatin changes in low cell number should enable the investigation of such questions.

The here-identified, novel role of *Rad21*/cohesin-mediated NF-κB signaling in impairing HSC self-renewal during aging could also be relevant for the selection of cohesin mutations in human aging and during early stage of myeloid leukemia. Cohesin mutations have been identified as rare mutation in aging (Jaiswal et al., 2014) and occur in 10–15% of myeloid leukemia (Kon et al., 2013; Thota et al., 2014), as well as in a variety of solid tumors (Barber et al., 2008; Guo et al., 2013). Early stages of tumor development are often associated with strong increases in inflammation/NF-κB signaling (Dolcet et al., 2005). The current study shows that *Rad21*/cohesin depleted HSCs are increasingly selected during aging. *Rad21*/cohesin suppression refers resistance to HSCs to undergo inflammation/NF-κB–induced differentiation and loss of self-renewal. The study shows that HSCs are more vulnerable than early progenitor cells (MPPs) to undergo aging/inflammation-induced increase in differentiation and loss of self-renewal. This increased sensitivity of HSCs associates with a more pronounced enhancement in ground-stage activity of NF-κB signaling and in failure to down-regulate inflammation/NF-κB signaling in the resolution stage of inflammation. It is tempting to speculate that the here described role of cohesin in mediating inflammation/NF-κB signaling, which impairs the self-renewal/expansion potential of HSCs during aging, could also represent a barrier for the expansion of cancer initiating cells. According to this model increases in inflammation signaling would be a relevant mechanism driving selection of *Rad21*/cohesin mutant cells at early stages of cancer development.

*Rad21*-mediated inflammation/NF-κB signaling could also contribute to aging-associated myeloid-skewed differentiation of the hematopoietic system. Our study shows that *Rad21*/cohesin deletion leads to increases in HSC self-renewal/expansion potential, especially in the context of aging and inflammation. Since, NF-κB signaling is required for the normal differentiation of HSPCs (Fang et al., 2018), it is possible that the selection of cohesin mutant HSCs with reduced sensitivity to respond to NF-κB signaling by itself contributes to the development of skewed differentiation. According to our study, cohesin mutant HSPCs exhibit increased HSC self-renewal coupled with impaired and myeloid-skewed differentiation, thus resembling two of the most prominent phenotypes of the aging hematopoietic system. It remains to be investigated in greater detail whether *Rad21*/cohesin is expressed at different levels in different subpopulations of HSCs (such My-biased vs. Ly-biased HSCs). If so, chronic increases in aging-associated inflammation may contribute to the selective expansion of HSC subpopulations with lower expression of *Rad21*/cohesin that are refractory to inflammation/NF-κB–induced differentiation. In support of this hypothesis, the current proteome experiment on HSCs shows that HSCs exhibit reduced RAD21/CTCF protein expression and increased CD150/VWF when comparing HSCs from old mice versus young mice. These data suggest that RAD21/CTCF may actually be expressed at lower levels in My-biased HSCs (with high expression of CD150/VWF), thus making this subpopulation more resistant to inflammation/NF-κB–mediated differentiation induction, which increases the selection of this subpopulation during inflammation and aging.

Altogether, the current study reveals mechanistic insights on how chronically activated inflammation and HSC-intrinsic changes in cohesin-dependent NF-κB signaling cooperate in promoting selection of HSCs with disrupted or reduced expression of *Rad21*/cohesin, increased self-renewal, and myeloid biased differentiation during aging.

## Materials and methods

### Mice

All wild-type mice were C57BL/6J mice and obtained from Janvier or from internal stock. NF-κB reporter and NF-κB/p50 knockout (*NF-κB1^−/−^*) mice were obtained from internal stock, and both mice were on a C57BL/6 background. Mice were maintained in a specific pathogen–free animal facility in Fritz Lipmann Institute with 12 h of light/dark cycle and fed with a standard mouse chow. Young and old mice were i.p. injected with LPS according to the body weight (1.5 mg/kg). Experiments were conducted according to protocols approved by the state government of Thuringia (reg. no. 03-006/13 and no. FLI-17-025).

### FACS analysis and sorting

For HSC, MPP and LSK cell isolation, c-Kit^+^ cells in total BM from mice were enriched with magnetic-activated cell separation (MACS; Miltenyi Biotec) according to the manufacturer’s protocol (LS Column, 130-042-402; anti–APC-microbeads, 130-090-855). Cells were incubated with a lineage cocktail containing biotinylated antibodies against CD4 (Biolegend; 100508), CD8 (Biolegend; 100704), TER-119 (Biolegend; 116204), CD11b (Biolegend; 101204), Gr-1 (Biolegend; 108404), and B220 (Biolegend; 103222) at 4°C for 30 min. After washing, cells were incubated with anti–CD34-FITC (eBioscience; 11-0341-85), anti–CD150-PE (Biolegend; 115904), anti–Sca-1-PE-Cy7 (Biolegend; 108114), and anti–c-Kit-APC (Biolegend; 105812) antibodies. Biotinylated antibodies were developed with streptavidin-APC-Cy7 (Biolegend; 405208). Cell sorting was performed using FACSAria III cell sorter. For peripheral blood (PB) analysis, blood was incubated with anti–CD45-PE (Biolegend; 103106), anti–CD4-APC (Biolegend; 100516), anti–CD8-APC (Biolegend; 100712), anti–Gr-1-APC-Cy7 (Biolegend; 108424), anti–CD11b-APC-Cy7 (Biolegend; 101226), anti–B220-APC (eBioscience; 17-0452-82), and anti–B220-APC-Cy7 (Biolegend; 103224) antibodies. Red blood cells were lysed with Lysis buffer (BD Biosciences; 555899) at room temperature for 5 min. For BM cell analysis, cells were incubated with a biotinylated lineage cocktail (4°C, 30 min), then incubated with anti–CD150-BV605 (Biolegend; 115927), anti–CD34-Alexa700 (eBioscience; 56-0341-82), anti–Sca-1-Pacific-Blue (Biolegend; 108120), anti–c-Kit APC (Biolegend; 105812), anti–IL-6R-PE (Biolegend; 115806), anti–TLR4-PE-Cy7 (Biolegend; 117610), and streptavidin-APC-Cy7 (Biolegend; 40520; 4°C, overnight). For cultured LSK cell analysis, cells were incubated with anti–CD48-APC (Biolegend; 103412), anti–Sca-1-PE (Biolegend; 108108), anti–c-Kit-PE-Cy7 (Biolegend; 105814), streptavidin-APC-Cy7 (Biolegend; 405208), lineage cocktail antibodies, or anti–CD11b-APC-Cy7 (Biolegend; 101226; 4°C, 30 min). For hematopoietic progenitor cell analysis, cells were incubated with anti–CD135(Flt3)-PE (Biolegend; 135306), anti–CD127(IL7Ra)-PerCP-Cy5.5 (Biolegend; 135022), anti–CD150-BV605 (Biolegend; 115927), anti–CD34-Alexa700 (eBioscience; 56-0341-82), anti–Sca-1-Pacific-Blue (Biolegend; 108120), anti–c-Kit APC (Biolegend; 105812), anti–FcγR III/II-PE-Cy7 (Biolegend; 101318), and streptavidin-APC-Cy7. FACS analysis was performed with BD LSRFortessa cell analyzer or FACSAria III cell sorter. Data were analyzed by FlowJo software. The gating strategy of hematopoietic stem and progenitors in BM and mature cells in PB is described in Fig. S5 (A and B).

### Lentivirus production

shRNA was inserted into the SF-LV-shRNA-EGFP plasmid using mir30 primers (Wang et al., 2012). Lenti-X (Clontech) cells were cultured in DMEM supplemented with 10% FBS, 100 U/ml penicillin, and 100 µg/ml streptomycin. Lentivirus was produced in Lenti-X cells using calcium phosphate transfection of 30 µg shRNA plasmid, 18 µg psPAX2, and 9 µg pMD2.G plasmids according to standard procedures (Schambach et al., 2006). Medium was changed 12 h after transfection and virus supernatant was collected 36 h after changing medium. Lentiviruses were concentrated at 106,800 rcf for 2.5 h at 4°C, and viral pellets were resuspended in sterile PBS. Luciferase shRNA: 5′-TGCTGTTGACAGTGAGCGCCCGCCTGAAGTCTCTGATTAATAGTGAAGCCACAGATGTATTAATCAGAGACTTCAGGCGGTTGCCTACTGCCTCGGA-3′; *Rad21* shRNA 1#: 5′-TGCTGTTGACAGTGAGCGCGCTTATAATGCCATTACTTTATAGTGAAGCCACAGATGTATAAAGTAATGGCATTATAAGCTTGCCTACTGCCTCGGA-3′; *Rad21* shRNA 2#: 5′-TGCTGTTGACAGTGAGCGCTTCGAGGATGACGACATGTTATAGTGAAGCCACAGATGTATAACATGTCGTCATCCTCGAAATGCCTACTGCCTCGGA-3′; *Rad21* shRNA 3#: 5′-TGCTGTTGACAGTGAGCGCTGGGACAAGAAGCTAACCAAATAGTGAAGCCACAGATGTATTTGGTTAGCTTCTTGTCCCAATGCCTACTGCCTCGGA-3′; *SMC1* shRNA: 5′-TGCTGTTGACAGTGAGCGACACAGGAGTATGACAAGCGAATAGTGAAGCCACAGATGTATTCGCTTGTCATACTCCTGTGCTGCCTACTGCCTCGGA-3′; *SMC3* shRNA: 5′-TGCTGTTGACAGTGAGCGCCAGCTTAGTGCTGAAAGACAATAGTGAAGCCACAGATGTATTGTCTTTCAGCACTAAGCTGTTGCCTACTGCCTCGGA-3′; *STAG1*(SA-1) shRNA: 5′-TGCTGTTGACAGTGAGCGACAGTGTTACAGGATTCAACTATAGTGAAGCCACAGATGTATAGTTGAATCCTGTAACACTGGTGCCTACTGCCTCGGA-3′; *STAG2*(SA-2) shRNA: 5′-TGCTGTTGACAGTGAGCGCCACAGCAGAGATGTTCAGACATAGTGAAGCCACAGATGTATGTCTGAACATCTCTGCTGTGATGCCTACTGCCTCGGA-3′.

### Cell cycle and apoptosis assay

Cell cycle analysis was performed via Ki67 staining (BD Biosciences; 556027) using Cytofix/Cytoperm kit (BD Biosciences; 554714). Apoptosis assays were performed by Annexin V Apoptosis Detection kit (BD PharMingen; 556547). Cell death was measured using FITC Annexin V apoptosis detection kit with 7-AAD (Biolegend; 640922).

### DR

Mice in the control group (AL) had unlimited access to standard food and the daily food intake was measured. DR mice were given 70% of the amount of the food consumed by AL mice. The food was given at certain time every day. DR started directly after the transplantation and lasted for 2 mo.

### Viral transduction of LSK cell

Freshly isolated LSK (3 × 10^5^) cells were plated in 400 µl serum-free expansion medium (SFEM; Stem Cell; 09650) with 100 U/ml penicillin, 100 µg/ml streptomycin, 50 ng/ml thrombopoietin (TPO; Peprotech; 315-14), and 50 ng/ml stem cell factor (SCF; Peprotech; 250-03) in a 48-well plate. Lentivirus suspensions were added into cells according to titration results. After 1–3 d, cultured LSK cells were collected and used for functional investigations.

### Transplantation assay

Viral-infected LSK cells were transplanted into lethally gamma-irradiated (12 Gy) recipient mice via i.v. injection. For the second round of transplantation, 5 × 10^6^ total BM cells from the primary recipient mice were serially transplanted into secondary recipient mice. After transplantation, recipient mice were treated with antibiotic water (0.01%; Baytril) for 1 wk and were monitored by weekly inspection until the end of the experiments. Chimerism and lineage composition of GFP^+^ cells in PB from recipient mice were periodically analyzed by FACS. At 4–9 mo after transplantation, chimerism and number of GFP^+^ HSCs in recipient mice were analyzed by FACS.

### Cell culture and inflammation treatment

Freshly isolated LSK cells were plated in 400 µl SFEM with 100 U/ml penicillin, 100 µg/ml streptomycin, 50 ng/ml TPO, and 50 ng/ml SCF in a 48-well plate, with or without LPS (200 ng/ml; Sigma; L2880) and Pam3CSK4 (1 µg/ml; InvivoGen; tlrl-pms) in the presence or absence of BMS-345541 (20 µM; Sigma; B9935). After 3–5 d culture, the number and percentage of HSPCs and differentiated cells were analyzed by FACS. 100 ng/ml of IL-6 (Peprotech; 216-16) was applied for IL-6 stimulation experiment.

### In vitro colony-forming assays

CD150^+^CD34^−^LSK cells were purified from the BM of 2–3-mo-old and 24-mo-old NF-κB reporter mice (*n* = 3 mice per age group) and seeded in methylcellulose medium (Stem Cell Technologies; M3434; 1.2 ml/well in 6-well plate) at 500 cells/triplicate (*n* = 3 biological repeats and 3 technical repeats). For serial round of plating, cells from the first plating were seeded at 5,000 cells/duplicate as above (*n* = 3 biological repeats and 2 technical repeats). Colony numbers were scored after 12 d in culture.

### IL-6 measurement

Young (3 mo old) and old (24 mo old) WT mice received an i.p. injection of LPS (1.5 mg/kg) and were sacrificed 3 h later. 5 × 10^6^ c-Kit^+^–enriched BM cells were isolated and cultured in 1 ml of SFEM with 100 U/ml penicillin, 100 µg/ml streptomycin, 50 ng/ml TPO, and 50 ng/ml SCF in a 24-well plate in the presence of secretion inhibitor Brefeldin A (BD Biosciences; 555029). After 4 h culture, cells were fixed and permeabilized using BD Fixation/Permeabilization Solution kit (554714). The level of IL-6 production in the HSC population was analyzed by FACS using IL-6 antibody (BD Biosciences; 554401).

### RNA-seq

RNA-seq was conducted on *Rad21* shRNA versus control shRNA-infected LSK cells that were either activated after a 3-d culture or reisolated from recipients, 4 mo after transplantation. All groups have samples in triplicate. Total RNA of cells was extracted using MagMAX-96 Total RNA Isolation kit (Ambion; AM1830) according to the manufacturer’s protocol. Sequencing of RNA samples was done using Illumina’s next-generation sequencing methodology (Bentley et al., 2008). In detail, quality check and quantification of total RNA was done using the Agilent Bioanalyzer 2100 in combination with the RNA 6000 nano kit (in vitro samples) or RNA 6000 pico kit (in vivo samples; Agilent Technologies Inc.). Library preparation was done using Illumina’s TruSeq RNA v2 library preparation kit (in vitro samples) or SMART-Seq v4 Ultra Low Input RNA kit (Takara Clontech) in combination with Illumina’s Nextera XT DNA library preparation kit (in vivo samples) following the manufacturer’s description. Quantification and quality check of libraries was done using the Agilent Bioanalyzer 2100 in combination with the DNA 7500 kit. Libraries were sequenced on a HiSeq2500 running in 51 cycle/single-end/high-output mode. In vivo as well as the in vitro samples were sequenced each in a single lane. Sequence information was extracted in FastQ format using Illumina’s bcl2fastq v1.8.4. Sequencing resulted in around 35 million reads per in vitro sample and around 31 million reads per in vivo sample.

### KEGG pathway analysis

RNA-seq data were initially quality controlled (FastQC; Andrews, 2010), depleted of remaining rRNAs (SortMeRNA; Kopylova et al., 2012), and quality trimmed (Prinseq; Schmieder and Edwards, 2011) before mapping to the mouse genome (GRCm38) with TopHat2 (Kim et al., 2013) and quantification by HTSeq-count (Anders et al., 2015) were performed. The DESeq2 (Love et al., 2014) pipeline was used to normalize read counts and to call significant DEGs. False discovery rate (FDR)–corrected P values were assumed to be significant at a threshold of 0.05. Further, DEGs were investigated for over-representation in mouse KEGG pathways by performing gene set enrichment analyses with the R-package GAUGE (Luo et al., 2009) using a hypergeometric test and FDR correction with a significance threshold of q < 0.1.

### ATAC-seq

LSK cells from 24-mo-old mice were infected with an shRNA against *Rad21* or a control shRNA (shLuci) and cultured for 2 d followed by an exposure to LPS/Pam3 (LPS: 200 ng/ml; Pam3: 1ug/ml) or PBS control for 14 h. At that time point LSK cells were subjected to ATAC-seq. ATAC-seq was performed with 50,000 cells as previously described (Buenrostro et al., 2015) with the following modifications: the transposition reaction was performed with 2 µl of Tn5 Transposase (Nextera; Illumina) for 1 h at 37°C. Transposed DNA was purified with the Monarch PCR Cleanup kit (NEB; T1030). Libraries were amplified with a total number of 14 cycles and purified with Agencourt AMPure XP beads (A63881). Large fragments were removed with a sample/bead ratio of 0.5 and remaining fragments were recovered with a ratio of 1.7. Sequencing of ATAC libraries was done using Illumina’s next-generation sequencing methodology (Bentley et al., 2008). Quantification and quality check of libraries were done using the Agilent Bioanalyzer 2100 in combination with the DNA 7500 kit. Libraries were pooled and sequenced on a NextSeq500 running in 40-cycle paired-end mode using sequencing chemistry v2. Sequence information was extracted in FastQ format using Illumina’s bcl2fastq v2.19.1.403. Sequencing resulted in around 43 million reads per sample. FastQ files quality check was performed with FastQC (v0.11.5). FastQ files mapping to mm9 genome was performed by using Bowtie (v1.1.2) with “best strata m 1” parameters. Duplicate reads were removed using a custom script. For peak calling, macs14 (v1.4.2) was used with “nolambda” parameter and two different P value cutoff (1e-3 and 1e-5). Peaks overlap with enhancers and promoters was done by using Bedtools intersect (v2.22.1). Other analyses were done by using R (v3.4.4). For finding the distribution of the peaks in the genomic regions, getTargetAnnotationStats and Intersect functions from Genomation package (v1.11.3) were used. For statistical analysis, peaks (1e-5) were merged using Peakreference function (TCseq_1.2.0 package). The merged peaks have used as the reference for calculation of read per million (RPM) for each sample by using custom script. RPM values for each peaks with interquartile ranges >1.5 was used for calculation of the Pearson correlation. Two-tailed *t* test was used to define the regions with significant changes in different comparisons (P value <0.05). Peaks enriched in shLuci (LPS/Pam3) versus shLuci (PBS) were defined as follows: P value <0.05, shLuci (LPS/Pam3)–RPM > shLuci (PBS)–RPM, and presence of the peak in both the replicate of shLuci (LPS/Pam3). Similarly, peaks depleted in shRad21 (LPS/Pam3) versus shLuci (LPS/Pam3) were defined as P value <0.05, shLuci (LPS/Pam3)–RPM > shRad21 (LPS/Pam3)–RPM, and presence of the peak in both the replicate of shLuci (LPS/Pam3). For motif analysis, the selected regions were intersected (Bedtools intersect) with the summit of the peaks in both replicates of shLuci (LPS/Pam3; Fig. 5, J and K) and the summits were used for motif discovery using Homer (findMotifsGenome.pl) with len: 4, 6, 8, 10, 12 and size: 100, 100 parameters. Differentially regulated peaks were associated to the nearest gene (ENSEMBLE annotation), whose transcription start site was ±5 kb or if overlapping with gene’s enhancer (as defined in GSE89767). Gene list and the log2 of RPM ratio was used for canonical pathway and upstream regulator analysis in Ingenuity Pathway Analysis (v45868156).

### Quantitative real-time PCR (RT-qPCR)

Total RNA was extracted using MagMAX-96 Total RNA Isolation kit (Ambion; AM1830) according to the manufacturer’s protocol. The GoScript Reverse Transcription System (Bio-Rad; 170–8891) was used for cDNA synthesis from total RNA according to manufacturer’s instructions. RT-qPCR was performed with an ABI 7500 Real-Time PCR System (Applied Biosystems). Amount of target RNA was normalized to that of the endogenous control β-actin (Actb). The gene expression quantities were determined according to the Pfaffl method with PCR efficiency corrections. Primers: Actb F: 5′-AAGGCCAACCGTGAAAAGAT-3′; Actb R: 5′- GTGGTACGACCAGAGGCATAC-3′; Ccl3 F: 5′-TTCTCTGTACCATGACACTCTGC-3′; Ccl3 R: 5′-CGTGGAATCTTCCGGCTGTAG-3′; Ccl4 F: 5′-TTCCTGCTGTTTCTCTTACACCT-3′; Ccl4 R: 5′-CTGTCTGCCTCTTTTGGTCAG-3′; Il-6 F: 5′-TGATGGATGCTACCAAACTGG-3′; Il-6 R: 5′-TTCATGTACTCCAGGTAGCTATGG-3′; Rad21 F: 5′-AGCAACCTGCACATGATGAC-3′; Rad21 R: 5′-ATAGTTGGCATCGGTTCGAC-3′; Il-1b F: 5′-AGTTGACGGACCCCAAAAG-3′; Il-1b R: 5′-TTTGAAGCTGGATGCTCTCAT-3′; GM-CSF F: 5′-GGCCTTGGAAGCATGTAGAA-3′; GM-CSF R: 5′-TCTGCACACATGTTAGCTTCTTG-3′.

### Western blot

5 × 10^5^ LSK cells were purified by FACS from young (2–3 mo old) and old (22–24 mo old) mice and lysed with 10 µl lysis buffer (50 mM Tris, pH 7.5, 150 mM NaCl, 1 mM EDTA, and 1% Triton X-100). The cells were sonicated (five cycles of 30 s on/30 s off) and clarified by centrifugation for 15 min (7,800 rcf; 4°C). The lysate (10 µg protein) was resolved through a 10% SDS-PAGE. The proteins were wet transferred using transfer buffer (20% methanol, 2.5 mM Tris, and 20 mM glycine) onto nitrocellulose membrane (Roth). The membrane was briefly washed with water (5 min), and equal total protein loading was ascertained by REVERT Total protein stain kit (LI-COR). The membrane was blocked in 4% nonfat milk in TBS buffer (150 mM NaCl and 50 mM Tris, pH 7.5) for 1 h and incubated overnight with anti–phospho-NF-κB p65 (Ser536; 1:1,000; Cell Signaling Technology; 3033) in 4% BSA in TBST buffer (TBS with 0.01% Tween-20). Consequently, the membrane was probed with IRDye 800CW-conjugated secondary antibody (1:10,000; LI-COR) dissolved in 4% milk in TBST buffer, and the images were obtained by Odyssey scanner (LI-COR).

### NanoString analysis

1 × 10^4^ of FACS-sorted HSCs (CD150^+^CD41^−^CD34^−^LSK) and MPPs (CD34^+^LSK) were lysed in 2 µl of lysis/binding solution (Applied Biosystems; 8500G14). The cell lysate was then used for hybridization reaction as follows: 2 µl of cell lysate was mixed with 5 µl of nCounter hybridization buffer (NanoString Technologies), 2 µl of Core Tagset, 2 µl of extension Tagset (NanoString Technologies), 0.5 µl of 0.6 nm Probe A working pool, 0.5 µl of 0.6 nm probe A extension Pool, 0.5 µl of 3 nm Probe B working pool, and 0.5 µl of 3 nm Probe B extension pool (IDT Technologies). 2 µl of nuclease-free water was added to each reaction to reach a final volume of 15 µl. The reaction mixture was prepared in Strip tubes (NanoString Technologies). Then, it was incubated at 67°C using thermal cycler (Masterycler; Eppendorf) for 16 h. Afterward, the NanoString chemistry was processed automatically using nCounter prep-station 5s (NanoString Technologies) according to manufacturer protocol. Directly after the run, the nCounter Cartridge was loaded into nCounter digital analyzer 5s (NanoString Technologies). Data analysis, heat map generation, and clustering were done after background correction using nSolver advanced analysis software (v4) and R software (v3.3.2). The following housekeeping genes were used for normalization: *ActB*, *B2M*, *Gapdh*, *Gusb*, *Hprt*, *PGK1*, *Polr1b*, *Polr2a*, *Ppia*, *Rpl19*, *Sdha*, and *Tbp*. For further information on sample preparation NanoString technologies, see https://www.nanostring.com/support/product-support/support-documentation.

### Proteomics of HSCs

In the first proteomic analysis, a total of 43 young (2–4 mo old) mice, 18 middle age mice (10–14 mo old), and 26 old mice (24 mo old) were used. 50,000 cells were isolated by pooling of HSCs (CD150^+^CD34^−^LSK) from different animals. In total *n* = 4 pools, *n* = 50,000 HSCs per pool from each age group were collected and analyzed. In the second proteomic analysis, a total of 36 young mice (6–8 mo old) and 10 old mice (30–36 mo old) were used. 100,000 cells were isolated by pooling of My-biased HSCs (CD150^hi^CD34^−^LSK) from different animals. In total *n* = 3 pools, *n* = 100,000 HSCs per pool from each age group were collected and analyzed. All mice used in both of the two proteomic analysis were C57BL/6J male mice. Experiments were analyzed by data-independent acquisition (DIA) mass spectrometry using a protocol optimized for low cells number. Cells were sorted directly into lysis buffer to a final concentration of 1% (wt/vol) SDS, 50 mM dithiothreitol, 100 mM Hepes, pH 8.0, and lysed by sonication using a Bioruptor Plus (Diagenode) with 10 cycles of 60 s on/30 s off at the highest setting at 20°C. Following reduction (10 min; 95°C) and alkylation (Iodoacetamide, 15 mM, 30 min, dark, room temperature), proteins were acetone precipitated and digested with 1:100 enzyme/protein LysC (Wako) for 4 h at 37°C in 100 mM Hepes, pH 8.0, containing 3 M urea and following 1:1 dilution with water to obtain 1.5 M urea, with 1:100 Trypsin (Promega) overnight at 37°C. Digested peptides were desalted using a Waters Oasis HLB µElution Plate. On reconstitution with 95:5 water: acetonitrile containing 0.1% formic acid to an estimated 1 µg/µl, peptides were spiked with retention time Hyper Reaction Monitoring kit (Biognosys AG) and analyzed on a Q Exactive HF-X (Thermo). Peptides were separated using the nanoAcquity MClass Ultra-High Performance Liquid Chromatography system (Waters) fitted with a trapping (nanoAcquity Symmetry C_18_, 5 µm, 180 µm × 20 mm) and an analytical column (nanoAcquity BEH C_18_, 1.7 µm, 75 µm × 250 mm). The outlet of the analytical column was coupled directly to the mass spectrometer using the Proxeon nanospray source. Solvent A was water and 0.1% formic acid, and solvent B was acetonitrile and 0.1% formic acid. The samples (∼1 µg) were loaded with a constant flow of solvent A at 5 µl/min onto the trapping column. Trapping time was 6 min. Peptides were eluted via the analytical column with a constant flow of 0.3 µl/min. During the elution step, the percentage of solvent B increased in a nonlinear fashion from 0–40% in 40 min (50,000 cell analyses)/120 min (100,000 cell analyses). The peptides were introduced into the mass spectrometer via a Pico-Tip Emitter 360-µm outer diameter × 20-µm inner diameter, 10-µm tip (New Objective), and a spray voltage of 2.2 kV was applied. The capillary temperature was set at 300°C. The radio frequency ion funnel was set to 40%. For data acquisition and processing of the raw data, Xcalibur 4.0 (Thermo) and Tune version 2.9 were used. For the DIA data acquisition full scan mass spectrometry (MS) spectra with mass range 350–1650 m/z were acquired in profile mode in the Orbitrap with resolution of 120,000. The default charge state was set to 3+. The filling time was set at maximum of 60 ms with limitation of 3 × 10^6^ ions. DIA scans were acquired with 22 (50,000 cells)/40 (100,000 cells) mass window segments of differing widths across the MS1 mass range. Higher collisional dissociation fragmentation (stepped normalized collision energy; 25.5, 27, and 30%) was applied and MS/MS spectra were acquired with a resolution of 30,000 with a fixed first mass of 200 m/z after accumulation of 3 × 10^6^ ions or after filling time of 40 ms (whichever occurred first). Data were acquired in profile mode. For sample-specific spectral library generation, representative samples of the different conditions were analyzed additionally by data-dependent acquisition, using the same gradients as for the DIA analyses. Both types of data were included in the library generation. The data were searched against the mouse Uniprot database (Swissprot entry only, release 2016_01; 16,747 entries) using the Pulsar search engine (Biognosys AG). Data were processed using Spectronaut 11 and Spectronaut Pulsar X Biognosys AG (Bruderer et al., 2017). Precursor matching, protein inference, and quantification were performed in Spectronaut using default settings, except that median intensities were used instead of Top3 to calculate protein quantities. Peptide and protein level FDR for DIA data were controlled to 1% (Rosenberger et al., 2017). Differential protein expression was evaluated using a pairwise *t* test performed at the precursor level, followed by multiple testing correction (Storey, 2002).

### Statistical analysis

Most figure panels contain data from experiments performed on the same day, except where stated when the cumulative data from multiple independent experiments were performed on different days. Numbers of biological replicates (independent samples, mice, or pools of mice) and experimental repetitions are stated in figure legends. The sample size used in each experiment was not predetermined by statistical power calculations. Blinding was not used in experiments. No animals were excluded from analyses. Mice were allocated to experiments randomly, and samples were processed in an arbitrary order, but formal randomization techniques were not used. Data were always presented as mean with SD, except where stated differently in figure legends. For analysis of the statistical significance of differences between two groups, we performed two-tailed Welch’s *t* test. To assess the statistical significance of differences between two treatments or conditions, we used two-way ANOVA followed by Tukey’s multiple comparison test. Where stated in figure legends, in the cases of cell number, ratio, or proportion data, we performed log transformation (for cell number and ratios) or logit transformation (for proportions) before statistical testing to approximate normal distribution with equal variances. The significance level was set at 0.05 (5%). All statistical analyses were performed with GraphPad Prism 7.01 software, except for regression analysis and the exhaustive pairwise comparisons, which were performed with r-project.org. Regression analysis was performed, minimizing the least squares error. The Z-test was used to test the difference in slope of the regression lines. The Wilcoxon test was used to test the difference in exhaustive pairwise comparisons.

### Data availability

The RNA-seq data have been deposited in the National Center for Biotechnology Information’s Gene Expression Omnibus and are accessible through Gene Expression Omnibus Series accession no. GSE110440. The ATAC-seq data discussed in this publication have been deposited in the National Center for Biotechnology Information’s Sequence Read Archive and are accessible through accession no. PRJNA501838. The mass spectrometry proteomics data have been deposited to the ProteomeXchange Consortium via the PRIDE partner repository (Vizcaíno et al., 2016; Deutsch et al., 2017) with the dataset identifier PXD011537.

### Online supplemental material

Fig. S1 shows the knockdown of *Rad21* enhances HSC self-renewal and impairs HSC differentiation, which is enhanced by DR. Fig. S2 shows knockdown of cohesin genes inhibits LPS/Pam3-induced differentiation of HSPCs, and shRad21-infected LSK cells show impaired activation of NF-κB target genes upon LPS stimulation. Fig. S3 shows inflammation-induced *Rad21*/NF-κB signaling limits the self-renewal of HSPCs during aging, which is rescued by *Rad21* knockdown or NF-κB inhibitor treatment. Fig. S4 shows LPS-induced inflammatory responses in hematopoietic cells of young and old wild-type mice. Fig. S5 shows gating strategies of FACS analyses.

## Supplementary Material

Supplemental Materials (PDF)
