# Supplementary material for: Cohesin-mediated NF-κB signaling limits hematopoietic stem cell self-renewal in aging and inflammation
Source: J Exp Med. 2019 Jan 7;216(1):152–75. doi: 10.1084/jem.20181505 (PMC6314529; doi:10.1084/jem.20181505)
Supplement: Supplemental Materials (PDF) [file JEM_20181505_sm.pdf]

JEM

## Supplemental material

Chen et al., https://doi.org/10.1084/jem.20181505

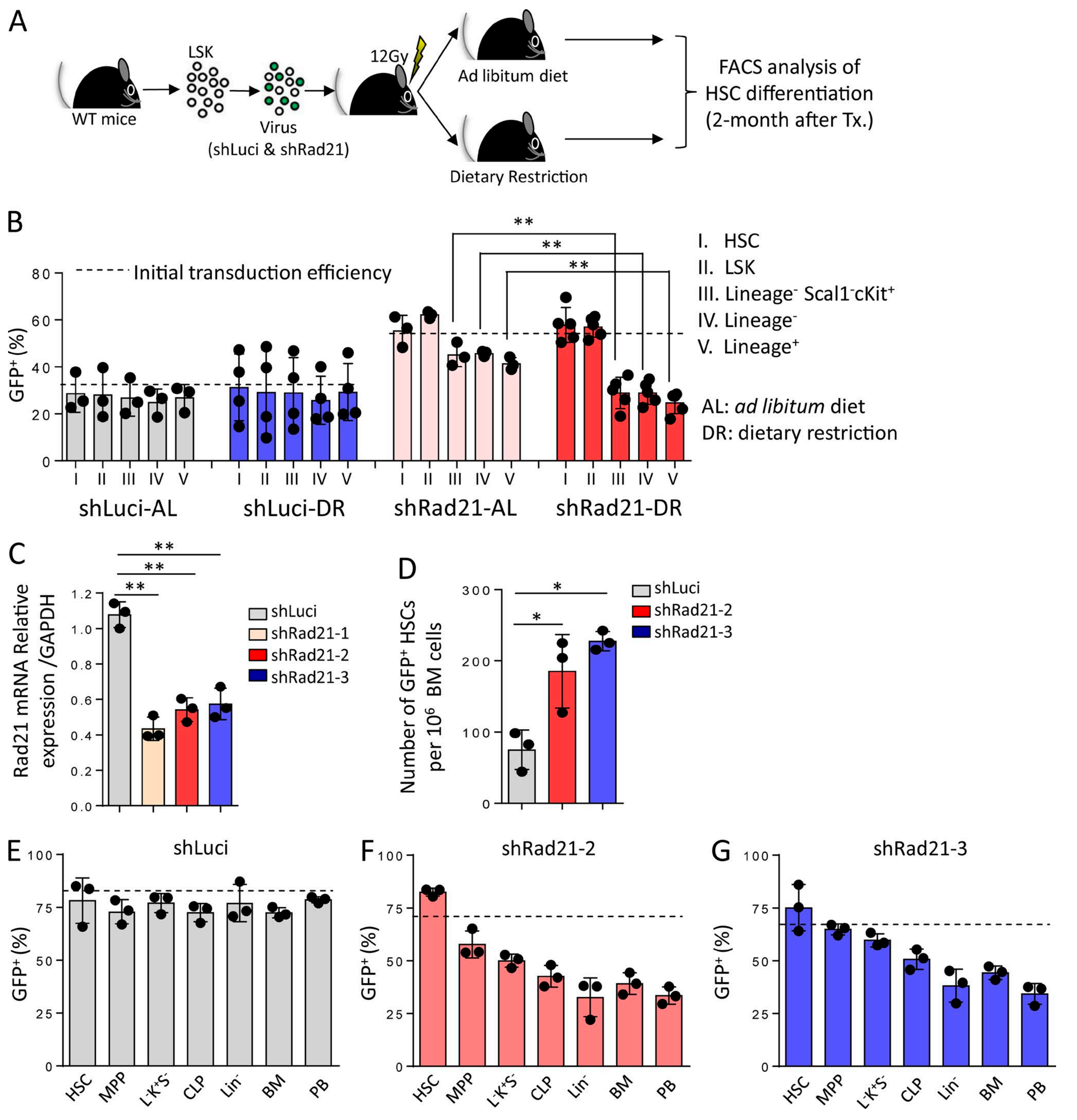


Figure S1. **Knockdown of *Rad21* enhances HSC self-renewal and impairs HSC differentiation, which is enhanced by DR. (A)** Schematic diagram of transplantation followed by 2-mo DR. LSK cells from 2-mo-old wild-type mice ($n$ = 10) were isolated and pooled. After viral transduction with shLuci or shRad21, transduced LSK cells (GFP positive) were transplanted along with nontransduced LSK cells (GFP negative) into recipient mice. Mice in the control group (AL diet) had unlimited access to standard food and the daily food intake was measured. DR mice were given 70% of the amount of the food consumed by AL mice. The initial transduction efficiency was 35% in shLuci AL and shLuci DR groups and 50% in shRad21 AL and shRad21 DR groups. **(B)** 2 mo after transplantation, percentages of donor-derived $GFP^+$ cells in indicated populations were analyzed by FACS. The dots represent individual mice (in total, $n$ = 3–5 recipient mice per group were analyzed in two independent experiments). Statistical significance was assessed with unpaired $t$ test with Welch's correction after logit transformation. **(C)** Three different *Rad21*-targeted shRNAs were checked for the knockdown efficiency in c-$Kit^+$ BM cells via qPCR (dots represents individual mice; $n$ = 3 biological repeats per shRNA). Statistical significance was assessed with unpaired $t$ test with Welch's correction. P values were calculated with Y = log(Y) transformation. **(D–G)** LSK cells from 2-mo-old wild-type mice were pooled, followed by shLuci, shRad21-2, or shRad21-3 virus transduction in culture and transplanted into lethally irradiated recipients. **(D)** The histogram depicts the number of $GFP^+$ HSCs 5 mo after transplantation. **(E–G)** The percentage of donor-derived $GFP^+$ cells in the indicated hematopoietic populations and groups was analyzed by FACS. The initial transduction efficiency was 79% for shLuci-LSK, 72% for shRad21-2-LSK, and 70% for shRad21-3-LSK. The dots represent individual mice ($n$ = 3 recipient mice per group). Statistical analysis was performed by unpaired $t$ test with Welch's correction on log-transformed data (D). All data represent mean ± SD; *, $P < 0.05$; **, $P < 0.01$. Tx, transplantation.

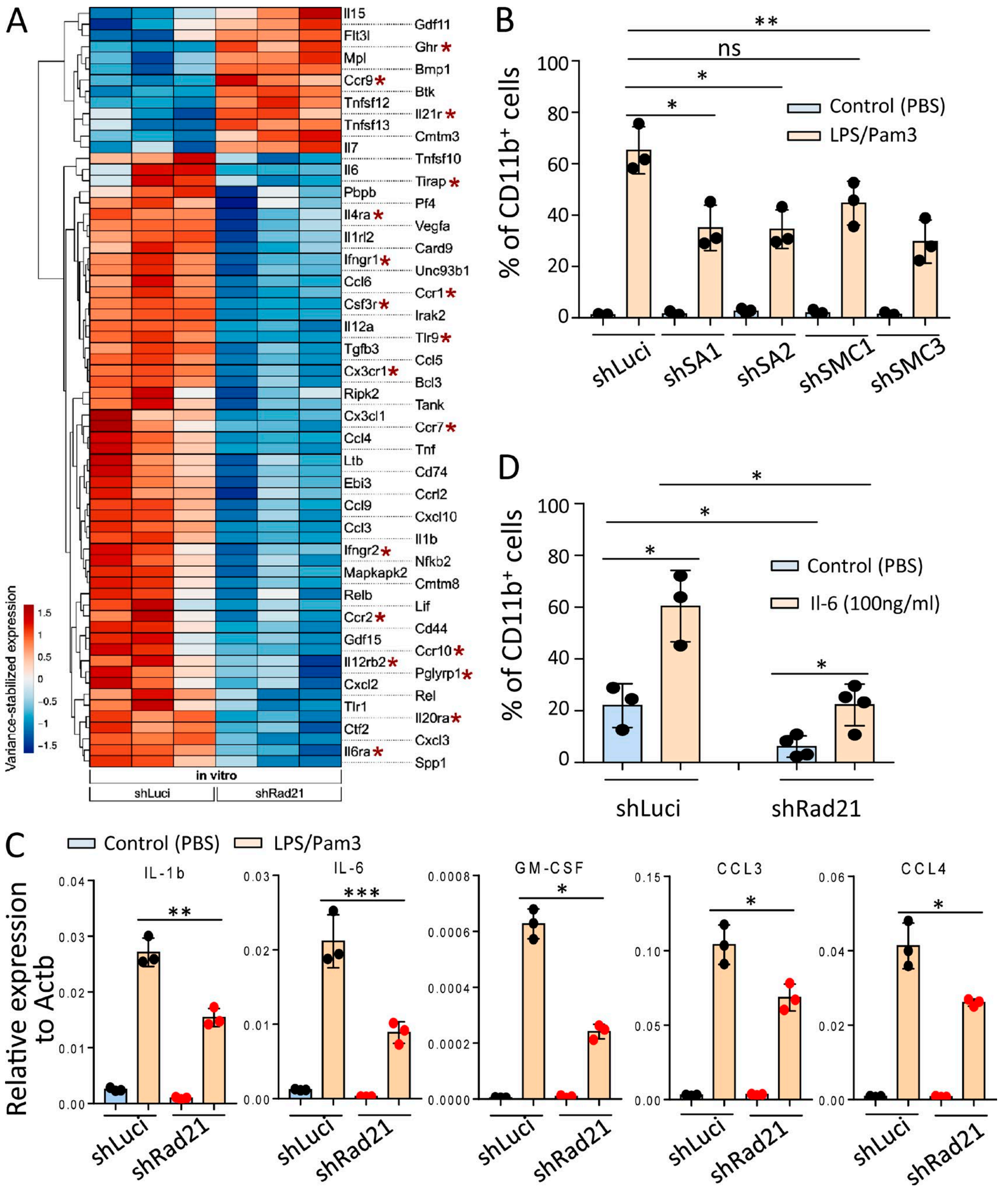


Figure S2. **Knockdown of cohesin genes inhibits LPS/Pam3-induced differentiation of HSPCs and *shRad21*-infected LSK cells show impaired activation of NF-κB target genes upon LPS stimulation. (A)** Analysis of DEGs in CD48⁻Sca-1⁺ cells purified from cultured LSK cells, 3 d after isolation and lentiviral targeting with shRNAs against *Rad21* (shRad21) or control (shLuci). Targeted cells (GFP⁺) were purified by FACS-sorting. The heat map shows DEGs that belong to pathways that were identified to be overrepresented in the list of DEGs comparing *Rad21* knockdown cells versus control cells by KEGG-pathway analysis (Fig. 4 A). The heat map shows row-scaled and variance-stabilized expression counts for each gene. Red asterisks highlight inflammatory receptor genes. **(B)** Freshly isolated LSK cells from 2-mo-old mice were lentivirally infected with an shRNA against *SA1*, *SA2*, *SMC1*, or *SMC3* or a control shRNA (shLuci). At 2 d after infection, cells were treated with LPS/Pam3 or PBS. The percentage of CD11b⁺ cells in the cultures was determined by FACS 3 d after LPS/Pam3 treatment. The dots represent individual mice ($n$ = 3 mice per condition). Statistical significance was assessed with two-way ANOVA followed by Tukey's multiple comparison test on logit-transformed data. **(C)** Freshly isolated LSK cells from 2–3-mo-old mice were lentivirally infected with an shRNA against *Rad21* or a control shRNA-luci. At 2 d after infection, cells were treated with LPS/Pam3 or PBS and analyzed 14 h later. The histograms show mRNA expression of the indicated NF-κB target genes relative to *Actb* in the indicated groups. The dots represent individual cultures derived from individual mice ($n$ = 3 mice per group). Statistical significance was assessed with two-way ANOVA followed by Tukey's multiple comparison test on log-transformed data. **(D)** LSK cells from young (2 mo old) donor mice were transduced with an shRNA against *Rad21* (shRad21) or a control shRNA (shLuci) and transplanted into young (2 mo old) recipient mice. 20 wk after transplantation, virally transduced (GFP⁺) LSK cells were isolated from the recipient mice that were transplanted with shLuci or shRad21 transduced HSPCs and cultured with or without IL-6 (100 ng/ml). Cultures were analyzed 4 d after initiation. The histogram shows quantification of the percentage of CD11b⁺ cells in the indicated groups in response to IL-6 supplementation or PBS control treatment ($n$ = 3–4 mice per group). Statistical significance was assessed with two-way ANOVA followed by Tukey's multiple comparison test on logit-transformed data. All data represent mean ± SD; *, P < 0.05; **, P < 0.01; ***, P < 0.001; ns, not significant.

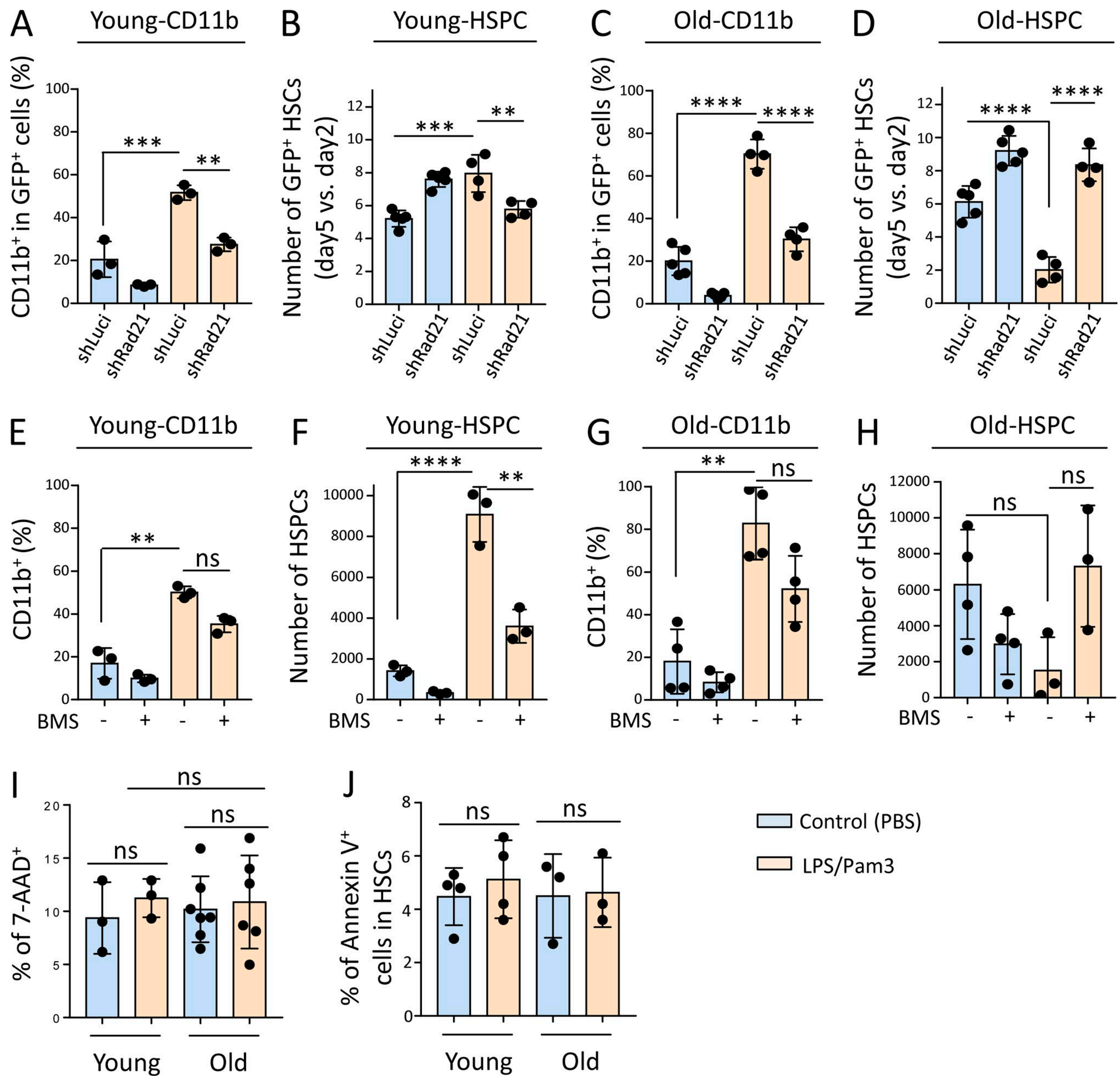


Figure S3. **Inflammation-induced *Rad21*/NF-κB signaling limits the self-renewal of HSPCs during aging, which is rescued by *Rad21* knockdown or NF-κB inhibitor treatment. (A–D)** Freshly isolated LSK cells from young (2 mo old) or old (24 mo old) mice were lentivirally infected with an shRNA against *Rad21* (shRad21) or a control shRNA (shLuci). At 2 d after infection, cells were treated with or without LPS/Pam3 and analyzed 3 d later. **(A and C)** Histograms show percentages of $CD11b^+$ cells in $GFP^+$ fraction of the cultures in the indicated groups. **(B and D)** Histograms show fold changes of the HSPC compartment ($CD48^-$LSK cells) relative to initial number of transduced $CD48^-$LSK cells as determined by FACS. The dots represent individual cultures from individual mice. (*n* = 3–5 mice per group). Statistical significance was assessed with two-way ANOVA followed by Tukey's multiple comparison test on logit-transformed data (A and C) or on log-transformed data (B and D). **(E–H)** 10,000 LSK cells from young (2 mo old) and old mice (24 mo old) were cultured with or without LPS/Pam3 treatment in the presence or absence of the NF-κB inhibitor, BMS-345541 (20 µM). At 5 d in culture, FACS analysis determined the percentages of $CD11b^+$ cells in the cultures from young (E) and old (G) mice and the absolute numbers of $CD48^-$LSK in the cultures from young (F) and old mice (H). The dots represent individual cultures (*n* = 3–4 mice per group). Statistical significance was assessed with two-way ANOVA followed by Tukey's multiple comparison test on logit-transformed data (E and G) or on log-transformed data (F and H). **(I)** LSK cells from young (2–3 mo old) and old (24 mo old) mice were cultured for 3 d with or without LPS/Pam3 treatment. The percentages of dead cells (7-$AAD^+$ cells) in the LSK cell cultures were determined by FACS. The dots represent individual mice. In total, *n* = 3 young and *n* = 6–7 old mice were analyzed in two independent experiments. **(J)** Young (2–3 mo old) and old (24 mo old) wild-type mice were injected with LPS via i.p. injection (1.5 mg/kg) and mice were sacrificed 24 h after injection. FACS analysis of apoptotic cells (Annexin $V^+$ cells) in HSCs of the indicated groups of mice. The dots represent individual mice. In total, *n* = 4 young mice and *n* = 3 old mice were analyzed in three independent experiments, and data were combined for analysis. **(E–I)** Statistical significance was assessed with two-way ANOVA followed by Tukey's multiple comparison test on logit-transformed data (E, G, I, and J) or on log-transformed data (F and H). All data represent mean ± SD; **, P < 0.01; ***, P < 0.001; ****, P < 0.0001; ns, not significant.

JEM

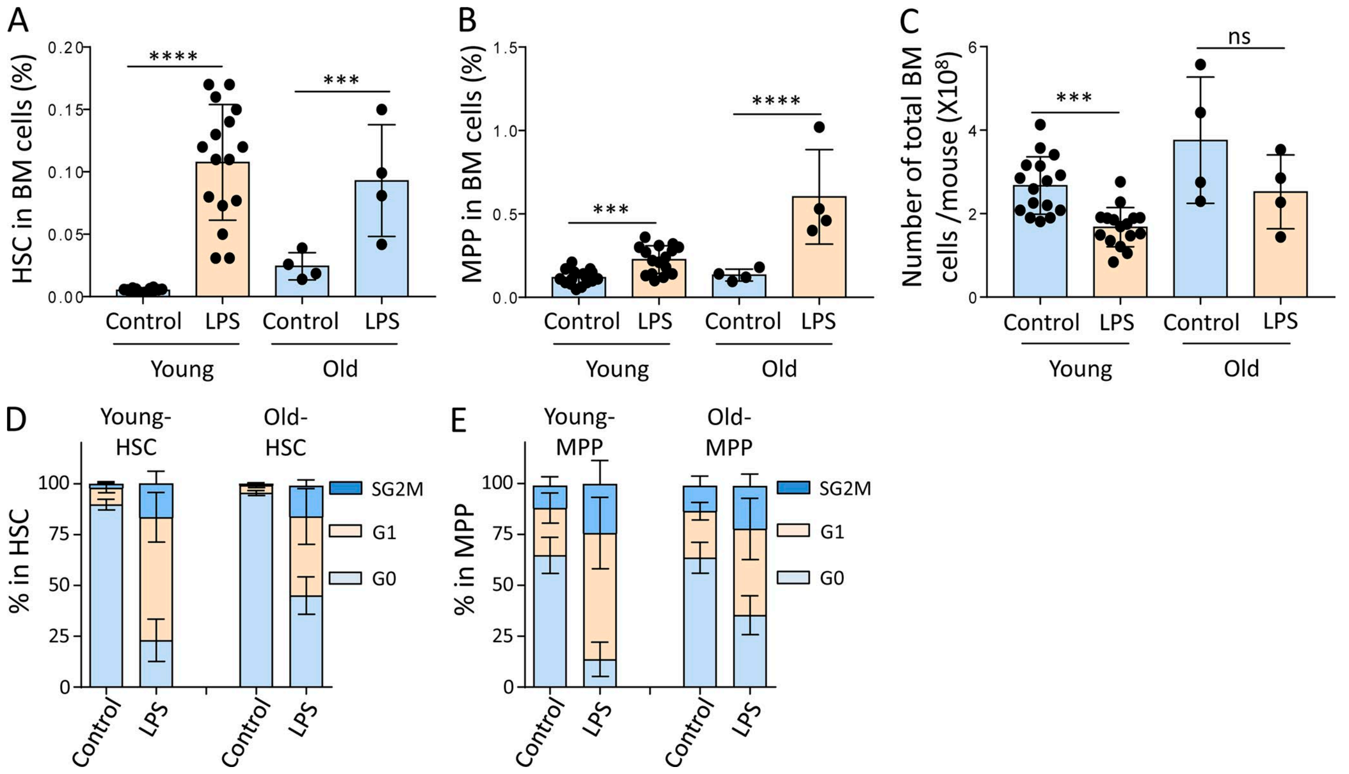


Figure S4. **LPS-induced in vivo responses of HSPCs in young and old wild-type mice. (A–E)** Young (2–3 mo old) and old (24 mo old) wild-type mice were injected with LPS via i.p. injection (1.5 mg/kg), and mice were sacrificed 24 h after injection. In total, $n$ = 16 young mice and $n$ = 4 old mice were analyzed in four independent experiments and data were combined for analysis. **(A and B)** The histograms show percentages of HSCs ($CD150^+CD41^-CD34^-LSK$) and MPPs ($CD34^+LSK$) in total BM cells of the mice in the indicated groups. The dots represent individual mice. Statistical significance was assessed with two-way ANOVA followed by Tukey's multiple comparison test on logit-transformed data. **(C)** The histogram depicts the total number of BM cells of the mice in the indicated groups. The dots represent individual mice. Statistical significance was assessed with two-way ANOVA followed by Tukey's multiple comparison test on log-transformed data. **(D and E)** FACS analysis of the cell cycle status of freshly isolated HSCs (D) and MPPs (E) of the indicated groups of mice. All data represent mean ± SD. ***, P < 0.001; ****, P < 0.0001; ns, not significant.

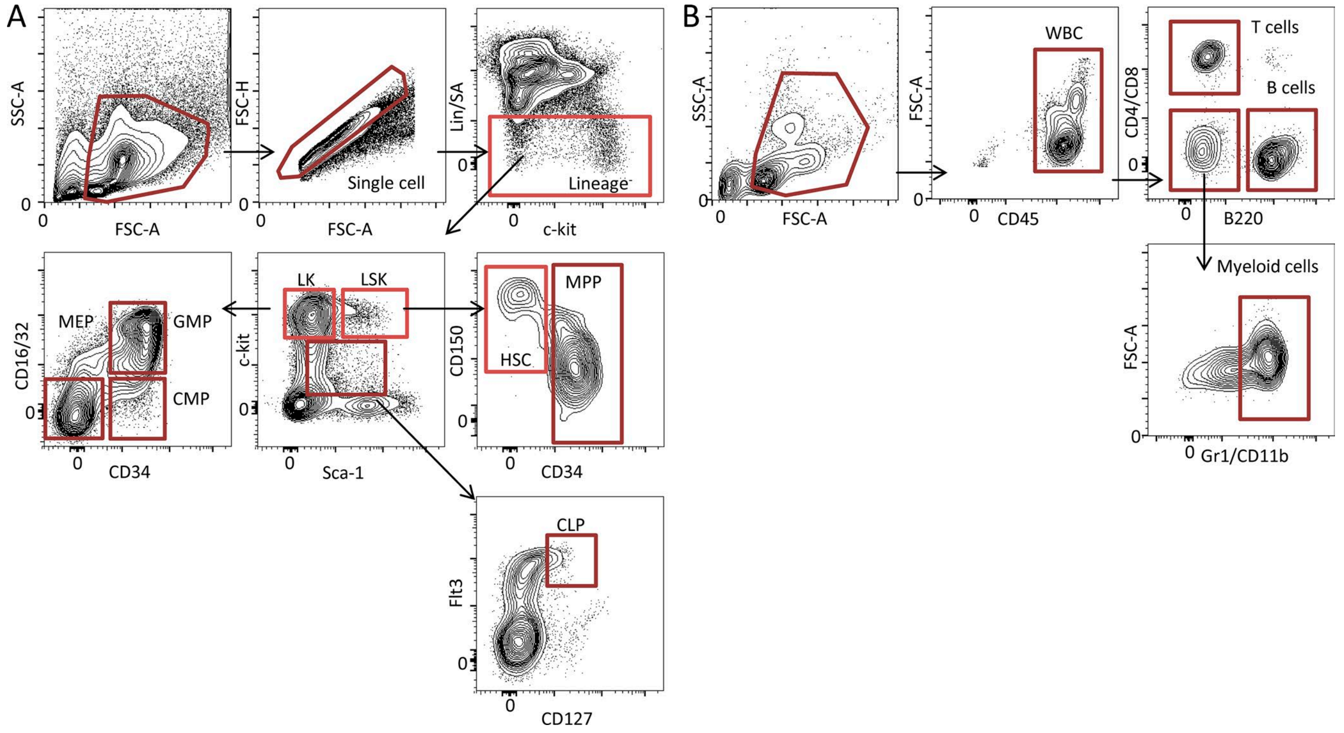


Figure S5. **Gating strategies of FACS analyses. (A)** Gating strategy of FACS analysis for HSPC populations in freshly isolated BM cells. **(B)** Gating strategy of FACS analysis for mature cell populations in PB cells. FSC, forward scatter; SSC, side scatter.
